# Live fast, die young and sleep later: Life history strategy and human sleep behavior

**DOI:** 10.1093/emph/eoaa048

**Published:** 2020-12-02

**Authors:** Vahe Dishakjian, Daniel M T Fessler, Adam Maxwell Sparks

**Affiliations:** 1 Department of Anthropology, University of California Los Angeles, Los Angeles, CA, USA; 2 Center for Behavior, Evolution and Culture, University of California Los Angeles, Los Angeles, CA, USA; 3 Bedari Kindness Institute, University of California Los Angeles, Los Angeles, CA, USA

**Keywords:** sleep, hedonic, life history theory, somatic maintenance, evolutionary medicine

## Abstract

**Background and objectives:**

Life History Theory (LHT) describes trade-offs that organisms make with regard to three investment pathways: growth, maintenance and reproduction. In light of the reparative functions of sleep, we examine sleep behaviors and corresponding attitudes as proximate manifestations of an individual’s underlying relative prioritization of short-term reproduction versus long-term maintenance.

**Methodology:**

We collected survey data from 568 participants across two online studies having different participant pools. We use a mixture of segmented and hierarchical regression models, structural equation modeling and machine learning to infer relationships between sleep duration/quality, attitudes about sleep and biodemographic/psychometric measures of life history strategy (LHS).

**Results:**

An age-mediated U- or V-shaped relationship appears when LHS is plotted against habitual sleep duration, with the fastest strategies occupying the sections of the curve with the highest mortality risk: < 6.5 hr (short sleep) and > 8.5 hr (long sleep). LH ‘fastness’ is associated with increased sleepiness and worse overall sleep quality: delayed sleep onset latency, more wakefulness after sleep onset, higher sleep–wake instability and greater sleep duration variability. Hedonic valuations of sleep may mediate the effects of LHS on certain sleep parameters.

**Conclusions and implications:**

The costs of deprioritizing maintenance can be parameterized in the domain of sleep, where ‘life history fastness’ corresponds with sleep patterns associated with greater senescence and mortality. Individual differences in sleep having significant health implications can thus be understood as components of lifelong trajectories likely stemming from calibration to developmental circumstances. Relatedly, hedonic valuations of sleep may constitute useful avenues for non-pharmacological management of chronic sleep disorders.

Lay Summary: Sleep is essential because it allows the body to repair and maintain itself. But time spent sleeping is time that cannot be spent doing other things. People differ in how much they prioritize immediate rewards, including sociosexual opportunities, versus long-term goals. In this research, we show that individual differences in sleep behaviors, and attitudes toward sleep, correspond with psychological and behavioral differences reflecting such differing priorities. Orientation toward sleep can thus be understood as part of the overall lifetime strategies that people pursue.

## INTRODUCTION 

Sleep, to a large degree, is nonnegotiable—yet we nonetheless often attempt to make bargains with it. We stay up too late, sleep in too long, or don’t sleep long enough, and then we pay a price—usually the next day, but sometimes much later down the line. Important as sleep may be for our health and well-being, other responsibilities and opportunities compete, sometimes successfully so, for our time and attention. Time is a resource that can never be replenished, and sleep sometimes demands more of it than we are inclined to spare. Metaphors for this tendency to barter with sleep can be found in our very phylogenetic history. Compared to our closest taxonomic relatives, we evolved to have the shortest sleep requirements, considerably less than the 10.3 hr that would otherwise be expected for a primate with our phenotypic characteristics [1]. But the advantages of reducing sleep in favor of sociability and predator and conspecific defense [1] may have been accompanied by a significant cost—our species’ unique susceptibility to Alzheimer’s disease [2]. In the world of natural selection, imperfection and compromise abounds. Evolved behaviors are subject to a host of constraints, trade-offs and opposing selective forces—and sleep is no exception.

Haig [3] argues that within the apparently serene nightscape of a mother sleeping beside her infant lies an ancient evolutionary tug-of-war: Paternally imprinted infant genes promote night-time waking and suckling behavior to enhance growth at the expense of the mother’s fertility, while maternally imprinted genes promote consolidated sleep in an effort to constrain the infant’s growth and shorten the mother’s inter-birth interval. Total domination by one side due to a chromosomal loss of function results in Prader–Willi (paternal deletion) or Angelman syndrome (maternal deletion), symptoms of which include excessive or fragmented sleep, respectively; normative development results from a delicate expressional stalemate between conflicting sets of genes [4]. Throughout *Homo sapiens’* evolutionary history, the intragenomic conflict over sleep is thought to have been influenced by local socioecological factors, such as sex-biased dispersal patterns, along with whether sleep was sacrificed altruistically (to protect the social group from danger) or for the purposes of increasing individual mating success [5].

Phylogenetics notwithstanding, sleep genotypes apparently interact with the environment to produce a wide range of phenotypes. In Hadza hunter-gatherers, for example, sleep–wake patterns demonstrate striking plasticity, changing flexibly in response to such environmental cues as activity level, light exposure, moon phase, day/night temperature and length of day [6]. Compared to Western populations, Hadza sleepers have stronger circadian rhythms, but also shorter and poorer quality sleep. At both the proximate and ultimate levels, human patterns of sleep tend to reflect a set of ecologically imposed demands and considerations.

Whereas a traditional medical view of sleep employs a largely mechanistic (pathophysiology) approach to understanding and treating sleep disorders, evolutionary perspectives can offer additional and complementary insights into the phylogenetic, ontogenetic and functional underpinnings of disordered patterns of sleep [7]. As with any health-related behavior that consists of both volitional and non-volitional aspects (e.g. eating), deviations from ‘normal’ sleep behavior may be subject to a host of folk explanations, in addition to complex social and cultural norms and sanctions, potentially resulting in the stigmatization of individuals with non-normative patterns of sleep. In this regard, functional and ontogenetic explanatory frameworks can act as non-judgmental substitutes for their lay equivalents. For health professionals, such frameworks may aid in empathizing with patients’ health-affecting habits and behaviors; the ability to effectively access another’s frame of reference is contingent upon first understanding it. Given that sleep is an evolved behavior, a full understanding of ‘disordered’ sleep necessarily includes an accounting of the various fitness costs and benefits that may be associated with a given pattern of sleep—both in relation to ancestral and modern contexts—along with the contributions of relevant neurobiological mechanisms, the individual life course, and interacting sociodemographic characteristics and experiences.

### A life history framework

Life History Theory (LHT) describes the fitness-relevant trade-offs among three investment pathways available to organisms: growth, maintenance and reproduction [8]. Because time, energy, and resources are limited, natural selection favors optimal allocational strategies that maximize fitness under environmental contingencies. For example, under conditions of high extrinsic mortality and resource scarcity, death may occur before long-term investments in growth and maintenance have paid off; hence, it pays to prioritize rapid maturation and reproduction over maintenance. Conversely, when the opposite patterns obtain, it pays to develop slowly in order to maximize phenotypic quality, the payoffs of which accrue over a long period of maintenance. While LHT was originally used to study interspecific differences in LH traits, it has since been also utilized to understand sources of individual variation [9].

According to the developmental plasticity model of LHT, features of the developmental environment which index likely characteristics of the adult environment calibrate lifelong patterns of prioritization in this regard, producing a probabilistically optimal LH strategy (LHS) along a ‘fast–slow’ continuum [10, 11]. Cues of environmental harshness and unpredictability promote the adoption of fast LH strategies, while inverse cues have the opposite effect [12]. These developmental ‘decisions’ require no conscious awareness; the timing of maturation and reproduction, and the overall trajectory, are informed by early hormonal responses to environmental features [13].

We aim to extend and corroborate the human LHT literature by linking measures of LH speed to assessments of sleep in order to parameterize maintenance investment—an understudied pathway in the context of human LH strategy.

### Sleep as a form of maintenance

The ultimate function of any maintenance process is to extend the period of reproductive effort (e.g. courting, mating, parenting)—either through diverting resources to critical systems (to facilitate short-term survival), or through repair processes (to facilitate long-term survival) [11]. Sleep constitutes investment in maintenance via mechanisms dedicated to restoration and regrowth [14, 15]. The maintenance functions of sleep occur predominately during the non-rapid eye movement (NREM) phase, which spans roughly 80% of the nightly sleep cycle in adults [15, 16]. The remaining time spent asleep occurs in the rapid eye movement (REM) phase, the function of which has yet to be conclusively determined [17]. Like other maintenance activities, sleep is largely mutually exclusive with both mating effort and parenting effort, the two forms of reproductive effort [5, 18]. The enormous time and opportunity costs of sleep hint at its relative paramountcy within the domain of maintenance, as sleeping precludes other vital somatic maintenance activities (foraging, eating, vigilance against predators and parasites, etc.).

Disrupted or altered sleep patterns entail maintenance costs in multiple ways. First, insufficient sleep duration can result in cardiovascular, metabolic and neurocognitive impairments [19, 20]. Second, excessive sleep duration is associated with increased risk of obesity, diabetes, cardiovascular disease, and stroke [21, 22]. Consolidating these findings, a U-shaped mortality curve is seen, with seven-hour sleepers at the curve’s nadir; habitual sleep durations shorter or longer than seven hours are associated with a dose-dependent increase in mortality risk [23–25]. Parallel U-shaped associations are found between sleep duration and inflammatory markers, with seven-hour sleepers showing the lowest levels of systemic inflammation; compared against short-sleepers, long-sleepers exhibit particularly elevated markers of inflammation [26, 27]. In experimental models, the release of pro-inflammatory cytokines following disrupted sleep [28] has been directly implicated in the pathogenesis of cardiovascular disease [29]. Thus, dysregulated innate immunity represents a plausible pathophysiological mechanism for the links between the extremes of sleep duration, chronic disease, and, ultimately, mortality [30, 31]. Duration notwithstanding, disturbed sleep (i.e. circadian instability, delayed sleep onset latency [SOL], and/or greater wakefulness after sleep onset [WASO]) is similarly associated with systemic inflammation and greater all-cause mortality [27, 32]—again hinting at the mediating role of innate immunity dysfunction [31].

### A proposed hedonic dimension to sleep

Complementing the above ultimate account of sleep, consider proximate mechanisms that may regulate sleep behavior. By analogy, note that feeding behavior is driven by both *homeostatic* (bioenergetic equilibrium) and *hedonic* (reward) mechanisms [33] that interact to regulate caloric intake [34]. Because adjustments to caloric intake can have downstream effects on maintenance processes [35], hedonic effects on appetite thus mediate the maintenance pathway. We postulate that, correspondingly, hedonic controls also mediate maintenance investments in sleep.

Hedonic *pleasure* describes positive affect acquired from the physical sensation of a behavior. With regard to sleep, this might include enjoyment derived from feelings of relaxation, comfort, and relief experienced in sleeping. Hedonic *motivation* describes the desire to engage in or avoid a behavior—dependent upon the conscious and unconscious reinforcers associated with said behavior [36]. This could include greater desire to engage in sleep after repeated positive reinforcement (e.g. greater energy and heightened cognitive alertness) following nights of quality sleep [37].

### Expression of sleep in the fast-slow LH continuum

A fast-LH strategy is ultimately defined by prioritization of current over future reproduction [11]. Proximately, this manifests as a phenotype characterized by the valuation of short-term gains at the expense of future rewards [38]. In the context of the nocturnal milieu, relevant short-term rewards include pleasure derived from night-time socialization and other actions that, via both direct and indirect pathways, would have resulted in reproduction in ancestral environments, while future rewards from quality sleep include positive affect, greater energy and good health in the near and distant future.

We hypothesize that, consciously or unconsciously, individuals weigh the hedonic rewards of habitual *non-sleep behavior* against the hedonic rewards of habitual *sleep behavior* when deciding how much to prioritize sleep. Given that the principal ultimate function of sleep is maintenance; that sleeping carries high opportunity costs; and that maintenance investments would be wasted by a lifespan suddenly cut short, we predict that, compared to slower-LH individuals, faster-LH individuals will have lower hedonic attributions of sleep—corresponding to a lower valuation of maintenance processes compared to alternative behaviors. Consequently, we predict that faster-LH individuals will experience measurable sleep–wake deficits and circadian disturbances. Providing preliminary grounds for this prediction, previous work documents associations between sleep variables and dimensions of impulsivity, with lower premeditation being very weakly associated with shorter sleep, and increased urgency (defined as the tendency to act rashly under extreme emotions) being associated with symptoms of insomnia [39]. In another study, interrelationships were found between short-term orientation, heightened sociosexuality and greater eveningness [40]. However, while including eveningness and sleep duration, our model goes on to predict that fast-LH individuals will display multiple manifestations of compromised sleep representing reduced investment into maintenance, including truncated sleep, delayed SOL, greater WASO, and/or circadian disruption by way of unstable sleep/wake times and varying sleep durations. In our preregistered initial formulation of our model, we thus expected a simple inverse relationship between LH speed and sleep duration. Subsequently, however, seeking to make sense of our results, we revisited the clinical literature, attending more closely to the documented detrimental correlates of *both* short sleep duration and long sleep duration. In contrast to our initial approach, our *post hoc* model therefore describes a U- or V-shaped relationship between LH speed and sleep duration.

To explore the associations between LH and sleep, we conducted two studies approved by the UCLA Office of the Human Research Protection Program. Measures used are reproduced in the Supplementary Data; registrations, datasets and analytic code are archived at https://osf.io/kgvyt/.

## Study 1 METHODS

### Participants

Two hundred and ninety-three adult participants were recruited via snowball sampling through the social networks of undergraduate research assistants not otherwise involved in the study. To maximize anonymity, no demographic information was collected. A single randomly selected participant was awarded a monetary prize, the amount and timing of which was determined by the participant’s responses to a discounting measure included in the survey. After filtering for a failed attention check or incompleteness, the final sample size was 263. Due to the nature of our recruitment protocol, it was not possible to calculate a response rate.

### Procedures and measures

#### LH strategy

There are two types of measures that can be used to describe individual variation in LH strategy—process (e.g. psychometric) and outcome (e.g. biometric/biodemographic) variables. Process variables attempt to measure LH allocational strategies by capturing the underlying latent variables (psychological processes) which presumably mediate clusters of ‘fast’ or ‘slow’ social and reproductive behavior. By contrast, outcome variables measure the ultimate objectives of those psychological processes—sexual onset, number of sexual partners, age of first parenthood, number of offspring, lifespan, and so on. Process and outcome variables each have trade-offs. Assuming that the construct validity of a psychometric LH measure has been established, the resulting process variable can allow for a convenient statistical description of the network of co-adapted attitudes, cognitions and psychosocial traits which might affect life history allocations in a fitness-increasing manner. In practice, attempting to compress all of individual LH variation into a single dimension may end up producing an overly facile depiction of a complex and multifarious entity [41].

An advantage of outcome variables is their ability to directly measure tangible, real-world correlates of LH, which process variables cannot do. On the other hand, outcome variables are prone to interpretive difficulties, often as a result of evolutionary disequilibrium. When the evolutionarily expected outcome of an evolved behavior is uncoupled from that behavior due to a mismatch between the environment of evolutionary adaptedness (EEA) and the current environment, the outcome variable may no longer accurately represent the proximate achievement of an ultimate objective. For example, widespread availability of contraception can uncouple the number of an individual’s offspring from the number of their sexual encounters—rendering changes in the meanings of both variables. To mitigate these limitations, we used a dual-pronged approach, employing both types of measures. Process and outcome variables are complimentary; the synthesis of the two can offer a unified perspective of events on the timeline of LH expression [42].

#### K-Factor

A previous factor analysis [43] of 20 theoretically specified LH subscales derived from items in the longitudinal MIDUS survey of health and well-being (*n* = 2095, Ages 25–74) revealed a general underlying factor (K-Factor) accounting for 70% of the reliable variance between cognitive and behavioral indicators of LHS; furthermore this latent factor was found to load alongside Covitality (Physical and Mental Functioning) and General Factor of Personality (a proposed social desirability factor derived from the ‘Big Five’ model of personality) on a single higher-order construct (Super-K) which explained 91% of the variance between the three lower-order factors, even after controlling for participants’ sex, race, self/spousal education and combined self/spousal/familial income from the previous 12 months. To measure the K-Factor, we used the Mini-K version of the Arizona Life History Battery; 20 items (in our work, α = 0.75) encompass six dimensions: insight, planning, and control; parental relationship quality; friend social contact/support; family social contact/support; pair-bonding; and community involvement. A meta-analysis of the Mini-K using data from over 30 studies and 7000 participants has shown high levels of nomological and convergent validity across a wide domain of constructs and measures conceptually related to future-oriented LH allocations, including: Physical and Mental Functioning, Romantic Partner Strategy, General Factor of Personality, Mutualistic Social Strategies, Antagonistic Social Strategies (reversed), Emotional Intelligence, Executive Functions, and Pro-Environmental Behavior [44]. A more positive K-Factor value indicates a slower-LH.

#### Sociosexual orientation

Sexuality is a central feature of LH strategy, hence, to measure mating orientation and previous sexual experience (PSE), we used an abbreviated version of the Multidimensional Sociosexual Orientation Inventory (MSOI) [45], which treats short-term mating orientation (STMO) (three items; α = 0.88) and long-term mating orientation (LTMO) (three items; α = 0.83) as separate dimensions. The PSE (three items; α = 0.77) asks participants their number of one-time and lifetime sexual partners, along with their number of sexual partners in the last year.

#### Temporal discounting

The human LH literature documents the developmental impact of childhood socioeconomic status and mortality cues on risk-taking behavior and impulsivity in adulthood [46]. Building on this, we presented participants with an abbreviated version of the Kirby *k* delay-discounting task (nine items), a measure of temporal discounting involving choosing between smaller near-term and greater long-term financial payments [47, 48]; participants were informed that one randomly selected participant would be paid according to one of their selections on this task. Higher *k* values represent steeper discounting of future rewards.

#### Sleep duration and quality

The Sleep Timing Questionnaire (STQ) [49] is a retrospective self-report measure of sleep in which habitual sleep duration is derived from multiple samplings of sleep and wake times (12 items; α = 0.95). Additionally, to gauge participants’ perceived sleep needs, we asked ‘How many hours of sleep do you need per night? (How many hours would you sleep if you could hypothetically sleep as long as you needed to?)’

#### Hedonic valuations of sleep

The hedonic value of sleep was assessed using two novel items. The *hedonic motivation* (HVS-M) item asks ‘If you could take a pill that would eliminate the need to sleep, how often would you take such a pill?’; response options on a five-point Likert scale are Never; Rarely; Regularly; Often; Always. The item was reverse coded, hence a more positive HVS-M value represents a higher hedonic motivation for sleep. The *hedonic pleasure* (HVS-P) item asks ‘Ignoring health considerations and biological requirements, how much do you enjoy the act of sleeping compared to other activities?’ Participants moved a slider (100 invisible graduations) ranging from ‘Sleeping is my least favorite activity’ to ‘Sleeping is my favorite activity.’ A more positive HVS-P value indicates greater hedonic pleasure from sleep.

#### Statistical analyses

All analyses were conducted using R (Version 3.6.1; R Core Team, 2019). To identify the relative contributions of LH strategies versus adjacent biodemographic outcomes with respect to sleep health, we applied hierarchical multiple linear regression models (see Supplementary Methods and Results). No variable exhibited serious multicollinearity, which was set at a threshold of variance inflation factor (VIF) > 10 [50].

While there is no consensus on precise definitions for ‘short sleep’ and ‘long sleep,’ Grandner [51] suggests that short sleep is commonly characterized as ranging from <6 hr to <7 hr of nightly sleep, while long sleep is typically defined as ranging from >8 hr to >9 hr. We chose the midpoints of these cutoffs, yielding <6.5 hr and >8.5 hr as the thresholds for ‘short’ and ‘long’ sleep, respectively. ‘Regular’ sleepers thus have habitual sleep durations ranging from 6.5 hr to 8.5 hr of nightly sleep.

### Results

#### Overview of key variables and interactions

The high correlation between lifetime sexual partners and sexual partners in the last year, *r*(261) = 0.98, *P* < 0.001, is consistent with the sample being composed primarily of young adults, as can be expected given our recruitment procedure. On average, participants reported needing 8.14 hr (SD = 1.19) of sleep per night, and an actual sleep duration of 7.44 hr (SD = 1.15). For a school or work day, the mean usual bedtime and waketime were 11:29 p.m. and 7:32 a.m., respectively. For a weekend/off day, the mean usual bedtime and waketime were 12:16 a.m. and 9:06 a.m. Correlations among major variables are reported in Table 1. See Supplementary Figs. S1 and S2 for qualitative responses regarding sleep detriments.

**Table 1.** Study 1: Life history, sociosexual orientation, and sleep-related variables: descriptive statistics and correlations with confidence intervals

Variable	*M*	*SD*	1	2	3	4	5	6	7	8	9	10	11
1. LH K-Factor^a^	1.22	0.67											
2. STMO^b^	3.54	1.92	−0.28^**^										
			(−0.38, −0.16)										
3. LTMO^b^	6.45	0.92	0.21^**^	−0.34^**^									
			(0.09, 0.32)	(−0.44, −0.22)									
4. Sex Partners	11.83	62.80	−0.13^*^	0.17^**^	−0.00								
			(−0.25, −0.01)	(0.05, 0.29)	(−0.12, 0.12)								
5. Eveningness	7.86	2.38	−0.07	0.19^**^	−0.01	−0.11							
			(−0.19, 0.05)	(0.07, 0.31)	(−0.13, 0.11)	(−0.23, 0.01)							
6. HVS-M^c^	3.66	1.01	0.07	−0.17^**^	0.02	0.09	−0.15^*^						
			(−0.05, 0.19)	(−0.28, −0.05)	(−0.10, 0.14)	(−0.03, 0.21)	(−0.26, −0.03)						
7. SWS^d^	7.80	2.10	0.14^*^	−0.11	0.09	0.09	−0.44^**^	0.27^**^					
			(0.02, 0.26)	(−0.23, 0.01)	(−0.03, 0.21)	(−0.03, 0.21)	(−0.54, −0.34)	(0.16, 0.38)					
8. SDV^e^	83.28	43.10	−0.10	0.06	0.03	0.10	0.22^**^	−0.15^*^	−0.11				
			(−0.22, 0.02)	(−0.06, 0.18)	(−0.09, 0.15)	(−0.02, 0.22)	(0.10, 0.33)	(−0.27, −0.03)	(−0.22, 0.02)				
9. H-SDV^f^	291.87	124.48	−0.16^**^	0.17^**^	−0.02	−0.15^*^	0.56^**^	−0.27^**^	−0.56^**^	0.43^**^			
			(−0.27, −0.04)	(0.05, 0.28)	(−0.14, 0.10)	(−0.27, −0.03)	(0.47, 0.64)	(−0.38, −0.15)	(−0.63, −0.47)	(0.32, 0.52)			
10. SOL^g^	24.86	24.22	−0.17^**^	0.00	−0.05	0.02	0.12	−0.10	−0.03	0.21^**^	0.15^*^		
			(−0.29, −0.05)	(−0.12, 0.12)	(−0.17, 0.08)	(−0.10, 0.14)	(−0.00, 0.24)	(−0.22, 0.02)	(−0.15, 0.09)	(0.09, 0.32)	(0.03, 0.26)		
11. WASO^h^	21.38	28.52	−0.15^*^	−0.02	0.06	0.17^**^	−0.13^*^	−0.02	−0.00	0.05	−0.07	0.30^**^	
			(−0.27, −0.03)	(−0.14, 0.10)	(−0.07, 0.18)	(0.05, 0.28)	(−0.25, −0.01)	(−0.14, 0.10)	(−0.13, 0.12)	(−0.07, 0.17)	(−0.19, 0.06)	(0.19, 0.41)	
12. Kirby *k*	0.01	0.03	−0.06	0.13^*^	−0.04	0.32^**^	−0.13^*^	0.03	0.06	0.10	−0.13^*^	0.06	0.18^**^
			(−0.18, 0.06)	(0.01, 0.25)	(−0.16, 0.09)	(0.21, 0.43)	(−0.25, −0.01)	(−0.09, 0.15)	(−0.06, 0.18)	(−0.02, 0.22)	(−0.25, −0.01)	(−0.06, 0.18)	(0.06, 0.30)

*M* and *SD* are used to represent mean and standard deviation, respectively. Values in square brackets indicate the 95% confidence interval for each correlation.

a LH K-Factor = Mini-K.

b STMO = short-term mating orientation, LTMO = long-term mating orientation.

c HVS-M = hedonic valuation of sleep (motivation).

d SWS = sleep–wake stability.

e SDV = sleep duration variability (minutes).

f H-SDV = hypothetical sleep duration variability (minutes).

g SOL = sleep onset latency (minutes).

h WASO = wake after sleep onset (minutes).

* *P* < 0.05.

** *P* < 0.01.

#### Are LH strategies instructive about participants’ habitual sleep durations?

**Figure 1.**
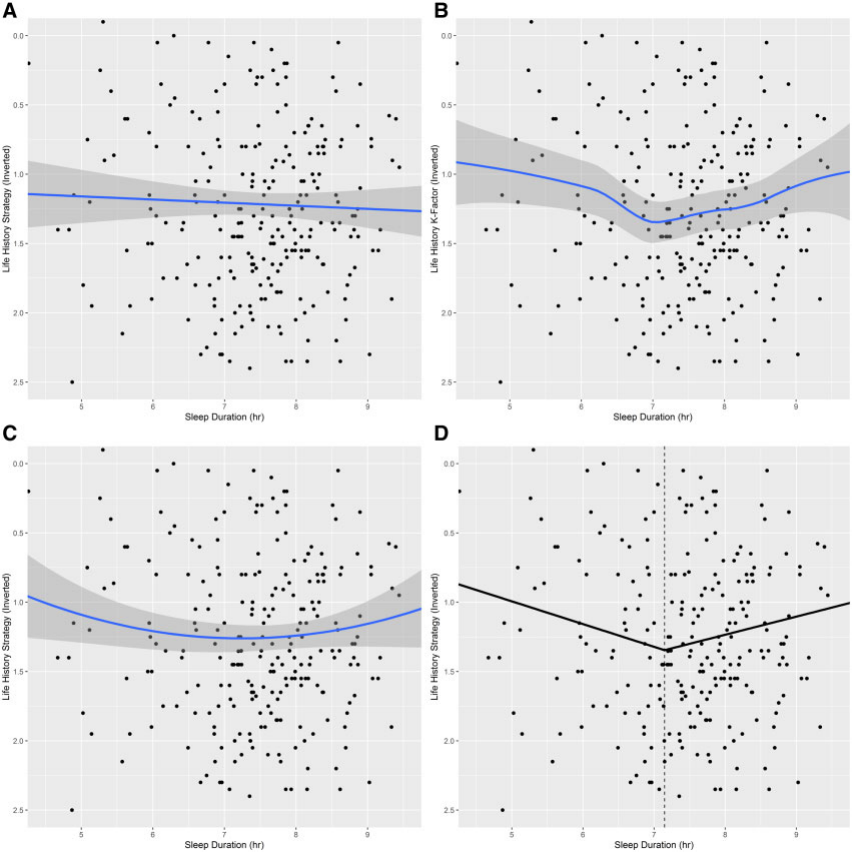
Study 1: Life history strategy versus habitual sleep duration. Values which are lower on the y-axis represent slower-LH strategies (higher K-Factor). Shaded regions represent the 95% confidence interval. (**A**) Null linear regression model. (**B**) LOESS (Locally Estimated Scatterplot Smoothing) model (span = 0.75). (**C**) Second order polynomial model; compared to the null model, this model had a significantly better fit (*P* = 0.042). (**D**) Segmented regression model; the dotted vertical line indicates the estimated breakpoint (7.15 hr). Compared to the null model, the breakpoint model had a significantly better fit (*P* = 0.029). R-squared values for the null, segmented, and 2nd order polynomial regression models were 0.001, 0.028 and 0.017, respectively. *n* = 263.

Employing a simple framing of sleep as involving a trade-off between sociosexual opportunities and maintenance, we initially operationalized our thesis as a linear prediction that faster-LH (lower K-Factor) individuals would sleep less than their slower-LH (higher K-Factor) peers. However, subsequent examination revealed that, congruent with the non-linear empirical relationships between sleep duration and mortality risk discussed earlier, three different modeling approaches support a non-linear interpretation of the relationship between K-Factor (LH-K) and sleep duration, with the steepest portion of the U- or V-shape (centered at roughly 7 hr) reflecting the slowest LH strategies (visualized in Fig. 1, model details in Supplementary Methods and Results).

An ANOVA between LH K-Factor and sleep duration category yielded a significant interaction effect, *F*(2, 260) = 4.25, *P* = 0.015. Contrasts revealed that, compared to sleepers in the regular range, habitual short-sleepers, *t*(2) = −2.58, *P* = 0.016 and long-sleepers *t*(2) = −2.91, *P* = 0.011 had significantly lower K-Factors (faster-LH strategies). When compared against long-sleepers, short-sleepers did not significantly differ in K-Factor, *t*(2) = −0.55, *P* = 0. 584. A Tukey’s honest significant difference test (HSD) confirmed a significant difference (*P* = 0.028) only between the K-Factor means of short (M = 1.01, SD = 0.77) and regular sleepers (M = 1.30, SD = 0.61), *d* = 0.45.

#### Do LH indicators interact with hedonic valuations of sleep behavior?

There was no significant linear relationship between LH-K and hedonic motivation (HVS-M), *r*(261) = 0.07, *P* = 0.246 or between LH-K and hedonic pleasure (HVS-P), *r*(261) = 0.02, *P* = 0.781 (non-linear analyses also did not yield convincing models of higher-order relationships; see Supplementary Methods and Results). However, a significant inverse correlation was found between short-term mating orientation and hedonic motivation (Table 1). Several significant yet weak relationships were observed between hedonic valuations of sleep and sleep behavior (Table 1); HVS-M was positively associated with sleep/wake stability (SWS) and negatively associated with eveningness, sleep duration variability (SDV), and hypothetical sleep duration variability (H-SDV). Moreover, HVS-M was shown to moderate the positive effect of LH-K on SWS (Supplementary Table S1). HVS-P was weakly but positively associated with self-reported sleep need, *r*(261) = 0.14, *P* = 0.024, time in bed on a weekend, *r*(261) = 0.19, *P* = 0.002, late-shifted time in bed on a weekend, *r*(261) = 0.25, *P* < 0.001, habitual time in bed, *r*(261) = 0.13, *P* = 0.031, habitual sleep duration, *r*(261) = 0.14, *P* = 0.021, and eveningness, *r*(261) = 0.15, P = 0.014. Finally, there was a significant positive correlation of 0.17 (*P* = 0.007) between hedonic motivation and hedonic pleasure.

#### Are LH indicators associated with sleep health?

Results demonstrated significant protective associations between LH slowness and sleep-wake stability (Supplementary Table S1), sleep onset latency (Supplementary Table S2), wakefulness after sleep onset (Supplementary Table S3), and hypothetical sleep duration variability (Supplementary Table S4)—even when controlling for mating effort. Higher temporal discounting rates were weakly associated with greater WASO, and very weakly negatively associated with both eveningness and H-SDV (Table 1). The combination of LH and sociosexual indicators explained between 2.2% (SDV) and 8.9% (eveningness) of the variance in parameters related to sleep health, and the full models including hedonic motivation and pleasure explained between 4.4% (SOL) and 14.6% (H-SDV) of the variance.

When our original simple prediction of an inverse linear relationship between LH speed and sleep duration is modified in light of the U-shaped relationship between sleep duration and mortality risk, the results of Study 1 can be understood as largely consistent with the hypothesized relationship between the prioritization of sleep and LHS. To examine the replicability of these results, in Study 2 we employed largely the same instruments with a different sample population.

Because Study 1 used a retrospective questionnaire of habitual sleep, these results are likely not reflective of day-to-day fluctuations in sleep duration. Since homeostatic mechanisms of sleep are fairly robust in ensuring that some lost sleep is eventually made up [52], a single value of habitual sleep duration might hold multiple meanings. For example, an individual who is perpetually sleep deprived during most of the week, but ‘catches up’ on the weekends [53], can have the same observed habitual sleep duration as someone who consistently sleeps the same number of hours per night, and experiences no transient sleepiness as a result. We therefore added a measure of participants’ sleepiness to address potential day-to-day fluctuations in sleep behavior.

## Study 2 METHODS

### Participants

Three hundred and eleven participants were recruited via Amazon’s Mechanical Turk platform. In addition to a randomly selected prize contingent upon selections made in the Kirby *k* measure as in Study 1, each participant was compensated $1.50 for the 15 min study. After applying the inclusion criteria used in Study 1, the final sample consisted of 305 adults (57% female), age 19–73 (M = 38.72 years, SD = 12.14).

### Procedures and measures

(For minor changes made to the sleep–wake stability and sleep-need items, see Supplementary Methods and Results.)

#### Hedonic motivation

The item used in Study 1 asks participants, ‘If you could take a pill that would eliminate the need to sleep, how often would you take such a pill?’ Out of concern that participants may have envisioned possible medical consequences, we added the following preface: ‘Scientists have invented a pill that temporarily eliminates the need to sleep for 24 hours. The pill does not permanently alter your body in any way, and has zero side effects.’

#### Sleepiness

To better capture individual variance in sleep behavior, we added the Karolinska Sleepiness Scale [54], a subjective measure of sleepiness.

#### Statistical analyses

A fundamental assumption of our model is that a fast life history strategy involves the pursuit of sociosexual behavior (investment in short-term reproduction) at the cost of sleep (investment in long-term maintenance). Single regression models—multivariate or otherwise—may confer information about the strength of relationships between variables, but they cannot unequivocally distinguish between downstream and upstream effects. We therefore used structural equation modeling, employing the LHT assumption that LH strategy is the biological antecedent to maintenance and reproduction-related psychological/behavioral outcomes (see Supplementary Methods and Results for details of model construction).

### Results

On average, participants reported that they needed 7.34 hr (SD = 1.16) of sleep per night, with an actual sleep duration of 7.32 hr (SD = 1.17). On school/work days, the usual mean bedtime and waketime were 11:00 p.m. and 7:06 a.m., respectively; on weekends/off days, these were 11:50 p.m. and 8:23 a.m., respectively. Correlations among major variables are reported in Supplementary Table S7. In Study 2, the mean temporal discounting rate was triple that of Study 1’s, while the SD was double. Additionally, the Kirby *k* showed no significant associations with any variable. See Supplementary Figs. S3 and S4 for qualitative responses regarding sleep detriments.

#### Does the association between LH ‘fastness’ and short/long sleep replicate?

**Figure 2.**
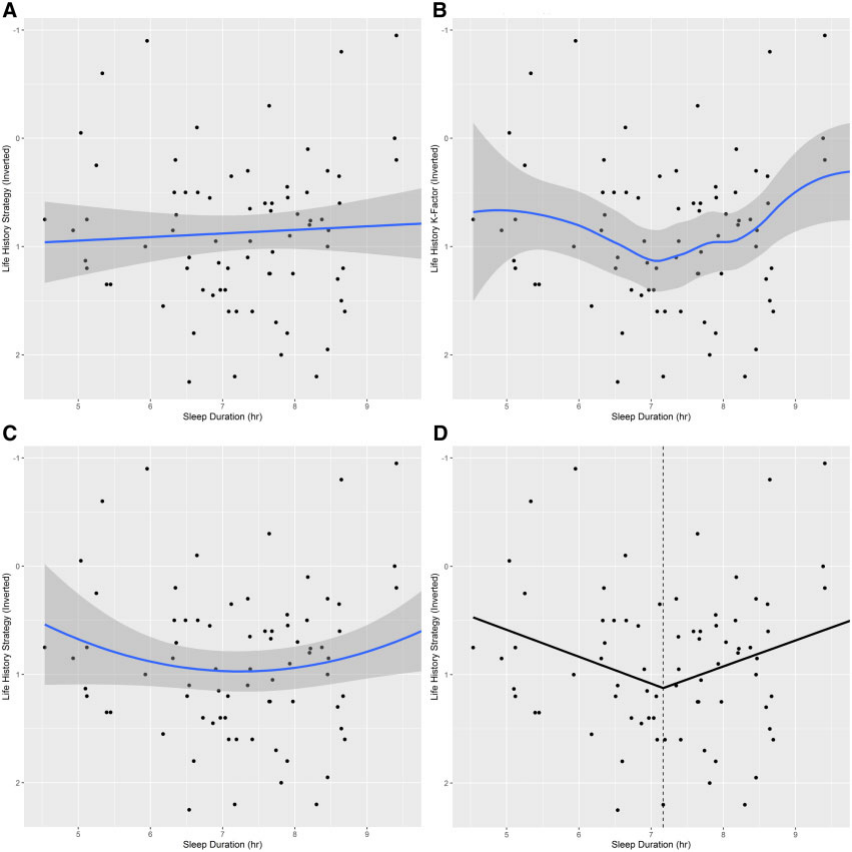
Study 2: Life history strategy versus habitual sleep duration (Aged 19–29). Values which are lower on the y-axis represent slower-LH strategies (higher K-Factor). Shaded regions represent the 95% confidence interval. (**A**) Null linear regression model. (**B**) LOESS (Locally Estimated Scatterplot Smoothing) model (span = 0.75). (**C**) Second order polynomial model; compared to the null model, this model had a significantly better fit (*P* = 0.045). (**D**) Segmented regression model; the dotted vertical line indicates the estimated breakpoint (7.17 hr). Compared to the null model, the breakpoint model had a significantly better fit (*P* = 0.040). R-squared values for the null, segmented, and 2nd order polynomial regression models were 0.004, 0.082, and 0.054, respectively. Aged 19–29 (M = 26.08, SD = 2.59). *n* = 79.

A central finding of Study 1 was that faster-LH (lower K-Factor) individuals are more likely to sleep less than, or more than, the optimal duration for health. When LH-K was again plotted against habitual sleep duration using LOESS, a U-shaped curve resulted (Supplementary Fig. S5), but with a slope that was gentler than Study 1’s. Similar to the plot in Study 1, the steepest portion of this fitted curve reflected the slowest LH strategies, with a segmented model converging on an estimated breakpoint of 6.85 hr (SE = 0.64). However, in Study 2, neither the breakpoint (*P* = 0.196) nor polynomial model (*P* = 0.162) achieved a significantly better fit than the null. To investigate whether this lack of significance was due to a Type 1 error in Study 1 or the result of a change in the sample population, we also conducted secondary analyses of the relationships between LH-K and habitual sleep duration in a younger subsample (Aged 19–29, M = 26.08, SD = 2.59) with the goal of approximating the presumed sample population of Study 1 while still maintaining a reasonable sample size (*n* = 79). Breakpoint analysis for this subsample converged on an estimate of 7.17 hr (SE = 0.53); both the V-shaped segmented (*P* = 0.040) and U-shaped polynomial (*P* = 0.045) models achieved a significantly better fit than the null (Fig. 2; see Supplementary Methods and Results for details).

An ANOVA between LH K-Factor and category of sleep duration in the full cohort yielded a nonsignificant interaction effect, *F*(2, 302) = 0.87, *P* = 0.421. However, ANOVA between LH K-Factor and category of sleep duration in the younger subsample demonstrated a significant interaction effect, *F*(2, 76) = 4.44, *P* = 0.015. Contrasts revealed that, as in Study 1, habitual short-sleepers, *t*(2) = −2.91, *P* = 0.008 and long-sleepers *t*(2) = −2.86, *P* = 0.008 had significantly lower K-Factors (faster-LH strategies) than sleepers in the regular range. As in Study 1, short- and long-sleepers did not vary significantly in K-Factor amongst themselves, *t*(2) = −0.44, *P* = 0. 658. A Tukey’s HSD confirmed a significant difference (*P* = 0.041) between only the K-Factor means of long (M = 0.52, SD = 0.93) and regular (M = 1.05, SD = 0.61) sleepers. The K-Factor mean difference (*d* = 0.67) between short (M = 0.63, SD = 0.66) and regular (M = 1.05, SD = 0.61) sleepers did not reach significance (*P* = 0.075) as it did in Study 1, potentially due to the smaller sample size of the younger cohort. Despite the lack of significant interaction effect between LH-K and habitual sleep duration category in the full sample, smaller LH-K values (faster-LHS’s) were still significantly associated with greater sleepiness (Supplementary Table S7).

#### Do LH indicators interact with hedonic valuations of sleep behavior?

In Study 2, there was a significant linear correlation between LH-K and hedonic motivation, *r*(303) = 0.13, *P* = 0.022; however, the relationship between LH-K and hedonic pleasure was insignificant, *r*(303) = −0.04, *P* = 0.475. Additionally, there was a significant inverse correlation between short-term mating orientation and hedonic motivation (Supplementary Table S7). Several significant yet weak relationships were observed between hedonic valuations of sleep and sleep behavior (Supplementary Table S7); HVS-M was again positively associated with sleep/wake stability and negatively associated with eveningness and H-SDV. In Study 2, negative associations were also found between HVS-M and both SOL and sleepiness. For HVS-P, we found positive associations with self-reported sleep need, *r*(303) = 0.24, *P* = <0.001, usual sleep duration on a weekday, *r*(303) = 0.11, *P* < 0.047, and early-shifted sleep duration on a weekday *r*(303) = 0.13, *P* = 0.026. Finally, there was a significant positive correlation, *r*(303) = 0.20, *P* < 0.001, between hedonic motivation and hedonic pleasure.

#### Are LH indicators associated with sleep health?

Regression modeling demonstrated significant protective associations between LH slowness and sleepiness (Supplementary Table S8), sleep onset latency (Supplementary Table S9), and sleep duration variability (Supplementary Table S10)—even after controlling for mating effort, hedonic valuations of sleep, and demographic variables (age, income, etc.). The full models explained between 7.3% (SDV) and 21.2% (eveningness) of the variance.

**Figure 3.**
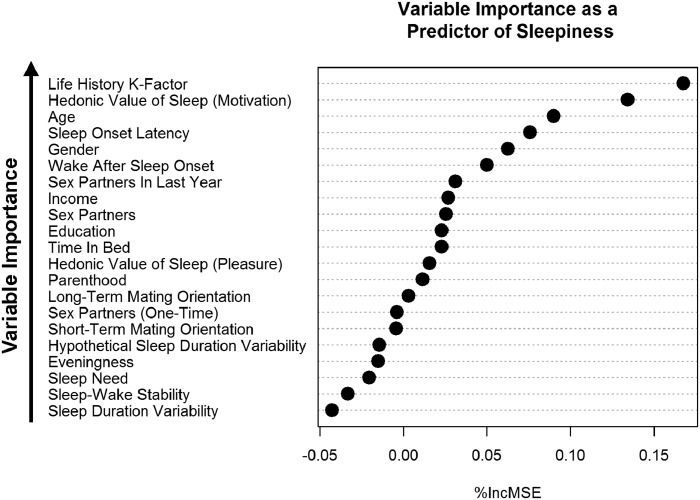
Study 2: Variable importance as a predictor of sleepiness using random forest decision trees. %IncMSE values are provided in raw, unstandardized form. A greater increase in mean square error after a variable is randomly permuted indicates greater importance as a predictor of sleepiness. Sleepiness was measured using the Karolinska Sleepiness Scale.

Employing a random forest machine learning model, we find that, among all measured variables (including demographics), LH-K was the single most important predictor of sleepiness (Fig. 3). In our full linear regression model, LH-K has the highest squared semi-partial correlation coefficient, with its significant effect remaining even after controlling for mating effort, hedonic valuations of sleep, and demographic variables (Supplementary Table S8)—further supporting LH-K’s importance as a predictor of sleepiness.

**Figure 4.**
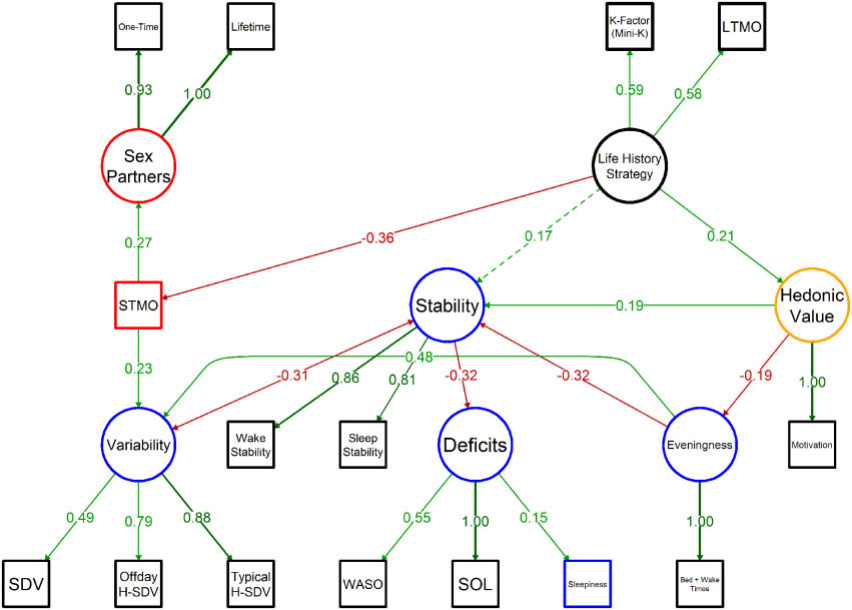
Study 2: Structural equation model of life history strategy’s effects on maintenance and reproduction allocations. Paths are labeled with standardized weights. Variables with a red border indicate investment into short-term reproduction, while variables with blue borders indicate investment into long-term maintenance. STMO is regressed (rather than loaded) on life history strategy, and sleepiness is regressed (rather than loaded) on deficits. Confirmatory factor analysis (CFA) was conducted using the full information maximum likelihood (FIML) estimator with robust (Huber–White) standard errors and a scaled test statistic that is asymptotically equivalent to the Yuan–Bentler test statistic. All solid paths are significant at *P* < 0.05, while dashed path coefficients have *P* values greater than 0.05. Comparative fit index (CFI) = 0.96, non-normed fit index (NNFI) = 0.94, goodness of fit index (GFI) = 0.99, root mean square error of approximation (RMSEA) = 0.06, standardized root mean square residual (SRMR) = 0.06. *n* = 305.

The structural equation model (SEM) is supportive of a positive effect of WASO and SOL on sleepiness, mediated by sleep–wake instability downstream of LH strategy (Fig. 4). Conceptually validating the LH measures, also downstream of LH strategy are negative effects on variables related to mating effort, with LH slowness being associated with lower STMO and, in turn, a lower number of sexual partners (both lifetime and one-time).

## DISCUSSION

### The maintenance costs of fastness

The primary function of sleep in adulthood is maintenance [15]. Sleep entails significant opportunity costs, notably in regard to sociosexual behavior. Life history involves trade-offs between maintenance and reproduction, hence individual variation in the relative prioritization of these two pathways should be reflected in sleep experience and valuation. Over 60 years’ worth of epidemiological studies reveal a U-shaped relationship between sleep duration and mortality, with the highest mortality risks seen in sleepers who diverge in either direction from 7 hr [55]. Here we find that when life history strategy is plotted against sleep duration, an analogous U-shaped curve results, with the fastest LH strategies occupying sections of the curve with the highest mortality risk. Interestingly, the strength of the non-linear association between LHS and sleep duration was mediated by age group, consistent with a possibly universal pattern [56] wherein youths and young adults forego sleep in favor of social activities—a pattern that may reflect fewer behavioral constraints placed by homeostatic mechanisms on young people than on older adults, as, due to senescence, the latter both have scarcer reserves for immediate use and recover more slowly from somatic insult.

In addition to issues of sleep duration, LH fastness was also associated with poorer quality sleep. Faster strategies were positively associated with wakefulness after sleep onset in Study 1; with sleepiness, sleep duration variability and eveningness in Study 2; and with sleep–wake instability, delayed sleep onset, and hypothetical sleep duration variability in both studies—all factors which may further compound the effects of short-sleeping on mortality. Although temporal discounting was linked to greater partner count and more WASO in Study 1, somewhat surprisingly, no association was found with LH-K; in Study 2, significant correlations were not found with any key variable. Responses on this measure may have been confounded by skepticism toward receipt of a long-term reward through the MTurk platform; additionally, results may reflect limitations of using delay-discounting tasks to measure delayed gratification, as these types of measures have been shown to conflate impulsivity with risk attitudes [57]. The latter construct varies in its relationship to LH strategy across domains, with financial risk attitudes showing very weak or null associations as compared to social, recreational, health/safety and ethical risk attitudes [58].

### Mechanisms of sleep disruption

The results of our hierarchical multiple regression models demonstrated significant positive associations between LH fastness and indicators of poor sleep quality, with multiple effects persisting even after controlling for sociosexual orientation, previous sexual experience, and parenthood. To wit, insofar as LH strategy can modulate sleep behavior, it may be able to do so through means which are independent of reproductive effort. Consilient with a LH framework of sleep, over two decades’ worth of longitudinal health and psychometric data from the MIDUS 2 project show direct paths between early life stress and adult trait anxiety, poor sleep quality, and lastly, physical health issues [59]. In our work, participants’ qualitative responses are suggestive of anxiety as having a discrete role in delaying the bedtimes of fast-LH individuals. In Study 1, only participants in the fastest tertile of LH strategy used the words ‘racing thoughts’ and ‘dreading’ to describe their greatest sleep impediments; over both studies, the word ‘anxiety’ was disproportionally used by faster-LH individuals (10-2). Heightened stress reactivity and greater vigilance in individuals with early life adversity—while maladaptive from a psychopathological perspective—may have adaptive function as a protective mechanism against future threat [60, 61].

In previous work, lower K-Factor scores (faster-LH strategies) were positively associated with stress, disruptive life events and maladaptive coping strategies; additionally, coping strategies and life events partially mediated the LH–stress association [62]. The effects of disruptive life events on sleep are well-established in studies examining racial discrimination in relation to racial/ethnic disparities in sleep outcomes; among both Maori New Zealanders and Black Americans, for example, the experience of racial discrimination was found to be an independent risk factor for sleep disturbance [63, 64]. Relatedly, in an 11-year longitudinal study of Black adolescent Americans, increased exposure to racial discrimination was positively associated with future indicators of LHS fastness in young adulthood [65]. This phenomenon may partially underlie previously documented differences in the manifestation of disordered sleep among American racial/ethnic minorities; while Black, Asian, and non-Mexican Hispanic/Latinx Americans are all more likely than non-Hispanic White Americans to exhibit short-sleep patterns [66], only Black Americans also display an increased likelihood of long-sleep, with this association remaining even after adjusting for sociodemographic factors (age, sex, income and geographic residence), body mass index, depression, functional capacity and medical illnesses [67]. Thus, racial discrimination may have compounding detrimental effects on sleep through its influence on multiple proximate pathways at different points across the life course.

Additionally, assuming that there is positive autocorrelation between developmental and adult environments, ‘fast’ surroundings (harsh and unpredictable) would by their very nature be antithetical to good sleep. In previous work, poor perceived neighborhood quality was associated with poorer sleep quality and health, with sleep quality partially mediating the association between neighborhood quality and health status [68]. Upregulated vigilance and psychological distress from the threat of neighborhood disorder and/or noise are detrimental to sleep quality [69, 70], and could reasonably explain patterns relating to short/disrupted sleep. Participants’ qualitative responses relating to their sleep detriments offer partial support for this notion, as, across both studies, ‘noise’ was disproportionally (12-2) noted by individuals in the lowest (fastest) tertile of K-Factor. Crucially, while stress, vigilance, and threat reactivity can all feasibly explain patterns related to truncated/disturbed sleep, none of the above mechanisms can provide a rationale for long/excessive sleep.

### Excessive sleep and LHS fastness

While we initially made no predictions about long-sleeping and LHS, this finding nevertheless can be understood as a deprioritization of maintenance among ‘fast’ sleepers, given that excessive sleep reflects underlying enhanced inflammatory responses [26, 27]. Acute inflammation, while central to the front-line defenses of innate immunity, is highly damaging on a chronic basis—contributing to senescence through physiological processes collectively referred to as ‘inflammaging’ [71]. Compared to acquired immunity, innate immunity is developmentally cheaper yet entails more collateral damage, hence extended reliance on innate immunity ultimately reflects relatively lower lifetime investment into maintenance [72, 73]. Hence, whereas the correlation between insufficient sleeping and inflammatory diseases may owe to the destructive consequences of chronically disrupted repair processes, the correlation between excessive sleeping and inflammatory diseases likely reflects differential investment in immune subsystems that prioritizes rapid development and reproduction over longevity. In terms of opportunity costs, long-sleeping is also congruent with a higher valuation of short-term sociosexual behavior; long-sleepers tend to be evening shifted [74], and the evening hours are when the overall desire for sex, and concomitantly, the number of sexual encounters, are at their highest [75]. Moreover, greater sociosexual behavior would presumably increase exposure to pathogens, which would in turn exacerbate the inflammatory burden. Thus, co-occurring predictive and state-based developmental plans [76] affecting multiple systems (e.g. immunological, neuropsychological) may reciprocally interact [77] to produce a ‘fast long-sleeping’ phenotype.

### Evidence for a hedonic dimension to sleep

The relationship between reward and sleep has previously been discussed primarily from the perspective of how sleep behavior is influenced by desire for behaviors which compete with sleep [78]. The action of sleeping itself is not typically considered to have its own hedonic component. In contrast, providing tentative internal validation of our constructs, in both studies we found that hedonic motivation for sleeping (HVS-M) was linked to sleep-related hedonic pleasure (HVS-P). Moreover, slower-LH strategies were weakly significantly associated with a greater hedonic motivation for sleeping in Study 2. Notably, in Study 1, HVS-M was negatively correlated with sleep duration variability; in Study 2, negatively correlated with SOL and sleepiness; and in both studies, negatively associated with eveningness, STMO, and hypothetical sleep duration variability. The lattermost nexus of replicated associations, given its constituent interrelationships, may be supportive of the hypothesis that hedonic signals of sleep compete with those related to night-time sociosexual activities; an alternative possibility is that night owls with short-term mating strategies go on to develop a lower hedonic motivation for sleeping after consistently experiencing worse quality and less refreshing sleep, since the relationship between hedonic stimuli and reward is reciprocal [79]. Altogether, individuals with a high hedonic motivation for sleep tend to engage in a healthier circadian pattern of sleep. A seemingly contradictory finding was the positive relationship between HVS-P and eveningness in Study 1. This may constitute a Type I error. Alternately, given evidence linking hedonism to eveningness [40], our HVS-P item may insufficiently differentiate sleep-derived hedonic pleasure from general hedonic pleasure. More intuitive were the positive relationships between HVS-P and weekend sleep duration, overall time spent in bed (Study 1), and weekday sleep duration (Study 2), suggesting that those who particularly enjoy the sensation of sleeping are more prone to lounging around in bed. Overall, HVS-M seems to be related to *better* sleep, while HVS-P appears to be related to *longer* sleep. Taken together, our results constitute preliminary evidence of the importance of sleep-specific hedonic dimensions in sleep behavior, independent of their mediating effects between LHS and sleep. The full extent to which hedonic facets of sleep control sleep behavior is unknown, as are their neurobiological correlates, calling for a fuller elucidation of these mechanisms in the future.

### Implications for evolutionary and integrative medicine

Evolutionary medicine holds that a full understanding of a given disease state requires an evolutionary exploration, i.e. mechanistic, functional, developmental and phylogenetic considerations of the underlying biological processes [80]. Absent these perspectives, a parochial view of disordered health—in which disease is simply regarded as a set of symptoms and associated mechanisms—can entail dire epidemiological costs [81]. In the U.S., acute sleepiness results in an estimated annual loss of 5000 lives, accompanied by 110 000 sleep-related injuries [82]; corresponding financial costs are in the dozens of billions [83]. This is to say nothing of the population-wide decreases in both lifespan and ‘healthspan’ due to chronic medical issues resulting from poor sleep [84]. Our work provides an instrumental evolutionary and ecological account of developmental processes that may contribute to sleep disorders, and sheds further evolutionary light on the avenues by which biopsychosocial factors during development can influence adult disease states [85]. Lastly, the role of hedonic reward as a potential mediator of sleep behavior may prove useful for non-pharmacological management of chronic sleep disorders.

### Limitations

Because our SEM model relies on cross-sectional data, and the time lags of hypothetical interactions between variables are non-instantaneous, although causation can plausibly be inferred, it cannot be proven [86]. Similarly, due to the inherently correlational nature of our study, we cannot rule out the possibility of a third intervening variable between LHS and associated sleep-related variables; at the same time, due to the conceptually meaningful network of both observed and previously documented interrelationships between our variables of study, such a hypothetical ‘third variable’, if found, would not necessarily be orthogonal with our existing interpretation of results (e.g. if physiological changes to the hypothalamic–pituitary–adrenal (HPA) axis or inflammatory markers were discovered to be the most proximal causative factors underlying the associations between LH fastness and short and long sleep, respectively, such findings would not fundamentally alter our central premise or attendant conclusions, and could instead plausibly represent extensions of the K-Factor’s nomological network). Caution is also warranted due to the potentially confounding influence of cultural norms, socioecological circumstances, and technology. Important differences in sleep likely occur between individuals in WEIRD societies [87] and individuals in societies in which technological control of the environment, the role of manual labor in production, and time spent outdoors are more characteristic of most of human history [88]. Cross-cultural investigations are thus needed to validate our results. Nevertheless, despite these limitations, we are encouraged by our positive findings given that there are *a priori* reasons to expect the postulated relationships to be noisy. While the fast–slow axis usefully describes LH covariation at both the between- and within-species levels, patterns between individuals are typically equivocated by the effects of individual stochasticity, differences in resource availability, and non-linear/interactive relationships between strategies and corresponding functional traits [89]. Furthermore, there may be additional between-individual LHS variation in the form of a separate ‘mating competition’ axis (e.g. mating effort, dominance seeking, etc.) [41], which may explain why long-term mating orientation loaded alongside the K-Factor in our SEM model, but short-term mating orientation did not. Our inclusion of the MSOI may have at least partially attenuated such a limitation by capturing a portion of the mating competition variance.

## CONCLUSION

Life History Theory describes a spectrum of trade-offs between growth, reproduction and maintenance, predicting that individuals will optimize investment in each in light of current and expected hazards and opportunities. Sleep, one of the principal avenues of maintenance, has high opportunity costs. Accordingly, we predicted that habitual sleep behavior would exhibit decrements or enhancements that correspond with overall life history strategies prioritizing mating effort over longevity or the converse, respectively. Consistent with this prediction, our findings suggest that faster life histories are associated with greater sociosexual behavior, poorer quality sleep, and sleep durations that are shorter or longer than optimal for health. Additionally, individuals with faster-LH strategies are more likely to suffer from acute sleepiness, which, together with poor quality sleep and extreme sleep durations, further heightens their associated mortality risk. Importantly, while fast-LH individuals do not seem to be aided by ultimate adaptations that reduce their objective sleep need, chronic curtailment may be facilitated by the subjective *perception* that sleep is relatively nonessential—perhaps via lower hedonic valuations for the action of sleeping. Thus, if the familiar exclamation, “I’ll sleep when I’m dead!” is intended to convey a sense of heedless bravado, our synthesis suggests that such a connotation may not only be earned, but dearly paid for, with never-ending sleep being tendered as final remittance.

## SUPPLEMENTARY DATA

Supplementary data is available at *EMPH* online.

## Supplementary Material

eoaa048_Supplementary_Data
