# Supplementary material for: Live fast, die young and sleep later: Life history strategy and human sleep behavior
Source: Evol Med Public Health. 2020 Dec 2;9(1):36–52. doi: 10.1093/emph/eoaa048 (PMC7953418; doi:10.1093/emph/eoaa048)
Supplement: eoaa048_Supplementary_Data [file eoaa048_supplementary_data.zip › EMPH-2020-089R2 Dishakjian et al Life History & Sleep Study 2-Survey Instruments-Corrected.pdf]

This questionnaire asks about when you normally sleep. We are interested in getting as accurate a picture as we can of the times when you normally go to bed and get up. Please think carefully before giving your answers and be as accurate and as specific as you can be.

Please answer in terms of a recent "normal average week," not one in which you traveled, vacationed or had family crises. Thank you.

Please think of GOOD NIGHT TIME as the time at which you are finally in bed and trying to fall asleep.

1. On the night before a work day or school day, what is your **earliest** GOOD NIGHT TIME? (e.g. 7:25 pm)

2. On the night before a work day or school day, what is your **latest** GOOD NIGHT TIME? (e.g. 7:25 pm)

3. On the night before a work day or school day, what is your **usual** GOOD NIGHT TIME? (e.g. 7:25 pm)

4. How stable (i.e., similar each night) are your GOOD NIGHT TIMES before a work day or school day? (select one)

	0-15 minutes	16-30 minutes	31-45 minutes	46-60 minutes	61-75 minutes	76-90 minutes	91-105 minutes	106-120 minutes	2-3 hours	3-4 hours	over 4 hours	
**within**	○	○	○	○	○	○	○	○	○	○	○	**of one another**

5. What are the three biggest factors that NEGATIVELY affect **(delay)** your GOOD NIGHT TIME before a work or school day? (you may leave one or more fields blank)

Largest Detriment

Moderate Detriment

Smallest Detriment

Night before a day off

This questionnaire asks about when you normally sleep. We are interested in getting as accurate a picture as we can of the times when you normally go to bed and get up. Please think carefully before giving your answers and be as accurate and as specific as you can be.

Please answer in terms of a recent "normal average week," not one in which you traveled, vacationed or had family crises. Thank you.

Please think of GOOD NIGHT TIME as the time at which you are finally in bed and trying to fall asleep.

6. On the night before a day off (e.g. a weekend), what is your **earliest** GOOD NIGHT TIME? (e.g. 7:25 pm)

7. On the night before a day off (e.g. a weekend), what is your **latest** GOOD NIGHT TIME? (e.g. 7:25 pm)

8. On the night before a day off (e.g. a weekend), what is your **usual** GOOD NIGHT TIME? (e.g. 7:25 pm)

9. How stable (i.e., similar each night) are your GOOD NIGHT TIMES on a night before a day off (e.g. a weekend)? (select one)

	0-15 minutes	16-30 minutes	31-45 minutes	46-60 minutes	61-75 minutes	76-90 minutes	91-105 minutes	106-120 minutes	2-3 hours	3-4 hours	over 4 hours	
**within**	○	○	○	○	○	○	○	○	○	○	○	**of one another**

10. What are the three biggest factors that NEGATIVELY affect **(delay)** your GOOD NIGHT TIME before a day off? (you may leave one or more fields blank)

Largest Detriment

Moderate Detriment

Smallest Detriment

Morning before a typical work or school day

This questionnaire asks about when you normally sleep. We are interested in getting as accurate a picture as we can of the times when you normally go to bed and get up. Please think carefully before giving your answers and be as accurate and as specific as you can be.

Please answer in terms of a recent "normal average week," not one in which you traveled, vacationed or had family crises. Thank you.

Please think of GOOD MORNING TIME as the time at which you finally get out of bed and start your day.

11. Before a work day or school day, what is your **earliest** GOOD MORNING TIME? (e.g. 7:25 am)

12. Before a work day or school day, what is your **latest** GOOD MORNING TIME? (e.g. 7:25 am)

13. Before a work day or school day, what is your **usual** GOOD MORNING TIME? (e.g. 7:25 am)

14. How stable (i.e., similar each morning) are your GOOD MORNING TIMES before a work day or school day? (select one)

	0-15 minutes	16-30 minutes	31-45 minutes	46-60 minutes	61-75 minutes	76-90 minutes	91-105 minutes	106-120 minutes	2-3 hours	3-4 hours	over 4 hours	
**within**	○	○	○	○	○	○	○	○	○	○	○	**of one another**

15. What are the three biggest factors that NEGATIVELY affect **(advance)** your GOOD MORNING TIME before a work or school day? (you may leave one or more fields blank)

Largest Detriment

Moderate Detriment

Smallest Detriment

Morning before a day off

This questionnaire asks about when you normally sleep. We are interested in getting as accurate a picture as we can of the times when you normally go to bed and get up. Please think carefully before giving your answers and be as accurate and as specific as you can be.

Please answer in terms of a recent "normal average week," not one in which you traveled, vacationed or had family crises. Thank you.

Please think of GOOD MORNING TIME as the time at which you finally get out of bed and start your day.

16. Before a day off (e.g. a weekend), what is your **earliest** GOOD MORNING TIME? (e.g. 7:25 am)

17. Before a day off (e.g. a weekend), what is your **latest** GOOD MORNING TIME? (e.g. 7:25 am)

18. Before a day off (e.g. a weekend), what is your **usual** GOOD MORNING TIME? (e.g. 7:25 am)

19. How stable (i.e., similar each morning) are your GOOD MORNING TIMES before a day off (e.g. a weekend)? (select one)

	0-15 minutes	16-30 minutes	31-45 minutes	46-60 minutes	61-75 minutes	76-90 minutes	91-105 minutes	106-120 minutes	2-3 hours	3-4 hours	over 4 hours	
**within**	○	○	○	○	○	○	○	○	○	○	○	**of one another**

20. What are the three biggest factors that NEGATIVELY affect **(advance)** your GOOD MORNING TIME before a day off? (you may leave one or more fields blank)

Largest Detriment

Moderate Detriment

Smallest Detriment

Unwanted Wakefulness and Attitudes Toward Sleeping

These questions are about how much sleep you lose to unwanted wakefulness.

21. On most nights how long, on average, does it take you to fall asleep after you start trying?

minutes

22. On most nights, how much sleep do you lose, on average, from waking up during the night (e.g. to go to the bathroom, to attend to a crying baby, because of noise from neighbors or cars)?

☐ minutes

23. Based on past experience, assuming you are not sleep deprived in any way, how many hours of sleep do you **need** per night in order to feel fully rested the next day?

I need ☐ hours and ☐ minutes of sleep per night.

24. Ignoring health considerations and biological requirements, how much do you enjoy the act of sleeping **compared to other activities**?

Sleeping is my least favorite activity — Sleeping is my favorite activity

25. Scientists have invented a pill that temporarily eliminates the need to sleep for 24 hours. The pill does not permanently alter your body in any way, and has zero side effects.

How often would you take such a pill?

Never	Rarely	Regularly	Often	Always
○	○	○	○	○

26. Here are some descriptors about how alert or sleepy you might be feeling right now. Please read them carefully and select the one that best describes how you feel at the moment.

Extremely alert	Very alert	Alert	Rather alert	Neither alert nor sleepy	Some sighs of sleepiness	Sleepy, but no difficulty remaining awake	Sleepy, some effort to keep alert	Extremely sleepy, fighting sleep
○	○	○	○	○	○	○	○	○

Mini-k (Part 1/2)

Please indicate how strongly you agree or disagree with the following statements.

27. I can often tell how things will turn out.

Disagree Strongly	Disagree Somewhat	Disagree Slightly	Don't Know/Not Applicable	Agree Slightly	Agree Somewhat	Agree Strongly
○	○	○	○	○	○	○

28. I try to understand how I got into a situation to figure out how to handle it.

Disagree Strongly	Disagree Somewhat	Disagree Slightly	Don't Know/Not Applicable	Agree Slightly	Agree Somewhat	Agree Strongly
○	○	○	○	○	○	○

29. I often find the bright side to a bad situation.

Disagree Strongly	Disagree Somewhat	Disagree Slightly	Don't Know/Not Applicable	Agree Slightly	Agree Somewhat	Agree Strongly
○	○	○	○	○	○	○

30. I don’t give up until I solve my problems.

Disagree Strongly	Disagree Somewhat	Disagree Slightly	Don't Know/Not Applicable	Agree Slightly	Agree Somewhat	Agree Strongly
○	○	○	○	○	○	○

31. I often make plans in advance.

Disagree Strongly	Disagree Somewhat	Disagree Slightly	Don't Know/Not Applicable	Agree Slightly	Agree Somewhat	Agree Strongly
○	○	○	○	○	○	○

32. I avoid taking risks.

Disagree Strongly	Disagree Somewhat	Disagree Slightly	Don't Know/Not Applicable	Agree Slightly	Agree Somewhat	Agree Strongly
○	○	○	○	○	○	○

33. While growing up, I had a close and warm relationship with my biological mother.

Disagree Strongly	Disagree Somewhat	Disagree Slightly	Don't Know/Not Applicable	Agree Slightly	Agree Somewhat	Agree Strongly
○	○	○	○	○	○	○

34. While growing up, I had a close and warm relationship with my biological father.

Disagree Strongly	Disagree Somewhat	Disagree Slightly	Don't Know/Not Applicable	Agree Slightly	Agree Somewhat	Agree Strongly
○	○	○	○	○	○	○

35. I have a close and warm relationship with my own children.

Disagree Strongly	Disagree Somewhat	Disagree Slightly	Don't Know/Not Applicable	Agree Slightly	Agree Somewhat	Agree Strongly
○	○	○	○	○	○	○

36. I have a close and warm romantic relationship with my sexual partner.

Disagree Strongly	Disagree Somewhat	Disagree Slightly	Don't Know/Not Applicable	Agree Slightly	Agree Somewhat	Agree Strongly
○	○	○	○	○	○	○

Mini-k (Part 2/2)

37. I would rather have one than several sexual relationships at a time.

Disagree Strongly	Disagree Somewhat	Disagree Slightly	Don't Know/Not Applicable	Agree Slightly	Agree Somewhat	Agree Strongly
○	○	○	○	○	○	○

38. I have to be closely attached to someone before I am comfortable having sex with them.

Disagree Strongly	Disagree Somewhat	Disagree Slightly	Don't Know/Not Applicable	Agree Slightly	Agree Somewhat	Agree Strongly
○	○	○	○	○	○	○

39. I am often in social contact with my blood relatives.

Disagree Strongly	Disagree Somewhat	Disagree Slightly	Don't Know/Not Applicable	Agree Slightly	Agree Somewhat	Agree Strongly
○	○	○	○	○	○	○

40. I often **get** emotional support and practical help from my blood relatives.

Disagree Strongly	Disagree Somewhat	Disagree Slightly	Don't Know/Not Applicable	Agree Slightly	Agree Somewhat	Agree Strongly
○	○	○	○	○	○	○

41. I often **give** emotional support and practical help to my blood relatives.

Disagree Strongly	Disagree Somewhat	Disagree Slightly	Don't Know/Not Applicable	Agree Slightly	Agree Somewhat	Agree Strongly
○	○	○	○	○	○	○

42. I am often in social contact with my friends.

Disagree Strongly	Disagree Somewhat	Disagree Slightly	Don't Know/Not Applicable	Agree Slightly	Agree Somewhat	Agree Strongly
○	○	○	○	○	○	○

43. I often **get** emotional support and practical help from my friends.

Disagree Strongly	Disagree Somewhat	Disagree Slightly	Don't Know/Not Applicable	Agree Slightly	Agree Somewhat	Agree Strongly
○	○	○	○	○	○	○

44. I often **give** emotional support and practical help to my friends.

Disagree Strongly	Disagree Somewhat	Disagree Slightly	Don't Know/Not Applicable	Agree Slightly	Agree Somewhat	Agree Strongly
○	○	○	○	○	○	○

45. I am closely connected to and involved in my community.

Disagree Strongly	Disagree Somewhat	Disagree Slightly	Don't Know/Not Applicable	Agree Slightly	Agree Somewhat	Agree Strongly
○	○	○	○	○	○	○

46. I am closely connected to and involved in my religion.

Disagree Strongly	Disagree Somewhat	Disagree Slightly	Don't Know/Not Applicable	Agree Slightly	Agree Somewhat	Agree Strongly
○	○	○	○	○	○	○

47. If you go outside on a clear day and look up, what color should the sky usually be?

Snack	Antelope	Pink	Blue	FM Radio	Tuberculosis	Printer
○	○	○	○	○	○	○

Multidimensional Sociosexual Orientation Inventory

48. I can easily imagine myself being comfortable and enjoying “casual” sex with different partners.

Disagree Strongly	Disagree Somewhat	Disagree Slightly	Don't Know/Not Applicable	Agree Slightly	Agree Somewhat	Agree Strongly
○	○	○	○	○	○	○

49. I can imagine myself enjoying a brief sexual encounter with someone I find very attractive.

Disagree Strongly	Disagree Somewhat	Disagree Slightly	Don't Know/Not Applicable	Agree Slightly	Agree Somewhat	Agree Strongly
○	○	○	○	○	○	○

50. I could easily imagine myself enjoying one night of sex with someone I would never see again.

Disagree Strongly	Disagree Somewhat	Disagree Slightly	Don't Know/Not Applicable	Agree Slightly	Agree Somewhat	Agree Strongly
○	○	○	○	○	○	○

51. I am interested in maintaining a long-term romantic relationship with someone special.

Disagree Strongly	Disagree Somewhat	Disagree Slightly	Don't Know/Not Applicable	Agree Slightly	Agree Somewhat	Agree Strongly
○	○	○	○	○	○	○

52. I hope to have a romantic relationship that lasts the rest of my life.

Disagree Strongly	Disagree Somewhat	Disagree Slightly	Don't Know/Not Applicable	Agree Slightly	Agree Somewhat	Agree Strongly
○	○	○	○	○	○	○

53. Long-term romantic relationships are not for me.

Disagree Strongly	Disagree Somewhat	Disagree Slightly	Don't Know/Not Applicable	Agree Slightly	Agree Somewhat	Agree Strongly
○	○	○	○	○	○	○

54. During your entire life, with how many partners have you had sexual intercourse?

☐

55. With how many partners have you had sexual intercourse within the past year?

☐

56. With how many partners have you had sex on one and only one occasion?

Email Address for Raffle

By participating in this survey, you have received a chance to win up to **$85**.

You will be asked to choose between prizes that vary in the amount and timing of payment. For example, you might be asked if you prefer to receive $1 tomorrow or $10 in one month. **The actual prize you receive, if you are chosen as the winner, will depend on these selections.** Should you win, you will receive instructions on how to claim your prize through a message attached to an MTurk worker bonus.

57. Please enter your MTurk **Worker ID**.

(Without your MTurk Worker ID, we can not deliver your prize, should you be chosen as the winner. Your Worker ID will **immediately be deleted** after a winner is chosen.)

Discounting

If you are selected as a winner, this prize will be randomly chosen from one of the following selections.

To make sure that you get a reward you prefer, **you should assume that you are the winner**, and then make each choice as though it were the one you will win.

Pick ONE from EACH possible choice.

58.

- $78 in the next 24 hours OR
- $80 in 162 days

59.

- $80 in the next 24 hours OR
- $85 in 157 days

60.

- $67 in the next 24 hours OR $75 in 119 days

61.

- $69 in the next 24 hours OR $85 in 91 days

62.

- $55 in the next 24 hours OR $75 in 61 days

63.

- $54 in the next 24 hours OR $80 in 30 days

64.

- $41 in the next 24 hours OR $75 in 20 days

65.

- $33 in the next 24 hours OR $80 in 14 days

66.

- $31 in the next 24 hours OR $85 in 7 days

Demographics

67. To which gender identity do you most identify?

- Female
- Male
- Transgender Female
- Transgender Male
- Other / Prefer Not to Say

68. What is your age, in years?

69. Are you the parent of at least one child who is currently age 16 or younger?

- Yes
- No

70. What is your height, in feet + inches (for example, 5 feet 4 inches)

Feet

Inches

71. Your political orientation?

Very Liberal	Somewhat Liberal	Slightly Liberal	Moderate	Slightly Conservative	Somewhat Conservative	Very Conservative

72. Your ethnicity (please choose closest)

- White / European
- Black / African American
- Asian / Asian American
- Hispanic / Latino
- South Asian
- Middle Eastern
- Native American
- Pacific Islander
- More than one
- Other

73. What is your approximate yearly household income in US dollars? (example: $29,000)

74. What is your highest level of education completed?

- Elementary / Middle School
- High School or equivalent
- Some college, no degree
- 2-year degree / certificate
- Bachelor's degree
- Some graduate / professional school
- M.A. / professional degree
- Ph.D. / M.D.

Thank You!
