# Supplementary material for: Live fast, die young and sleep later: Life history strategy and human sleep behavior
Source: Evol Med Public Health. 2020 Dec 2;9(1):36–52. doi: 10.1093/emph/eoaa048 (PMC7953418; doi:10.1093/emph/eoaa048)

**Figure S1. Study 1: Self-Reported Sleep Detriments for a Weekday Evening**

Word clouds were built from a free-response item asking participants about the biggest factor that delayed their bedtime. Larger words in word clouds indicate higher frequencies of word usage. Fast and Slow LH refer to the lowest and highest tertile of the sample LH K-Factor distribution as measured by the Mini-K scale. (a) Word cloud of Fast-LH participants’ responses (b) The ten most frequently used words in Fast-LH responses (c) Word cloud of Slow-LH participants’ responses (d) The ten most frequently used words in Slow-LH responses


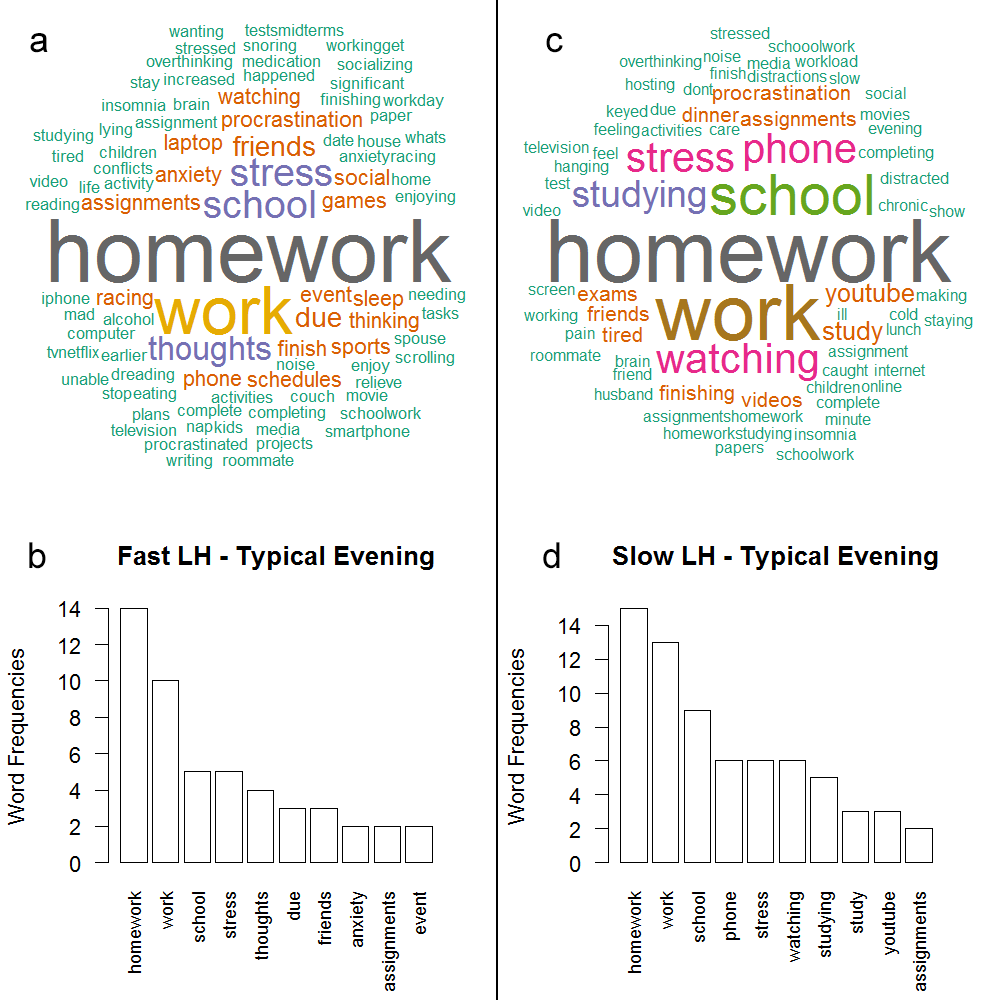


**Figure S2. Study 1: Self-Reported Sleep Detriments for a Weekend Evening**

Word clouds were built from a free-response item asking participants about the biggest factor that delayed their bedtime. Larger words in word clouds indicate higher frequencies of word usage. Fast and Slow LH refer to the lowest and highest tertile of the sample LH K-Factor distribution as measured by the Mini-K scale. (a) Word cloud of Fast-LH participants’ responses (b) The ten most frequently used words in Fast-LH responses (c) Word cloud of Slow-LH participants’ responses (d) The ten most frequently used words in Slow-LH responses


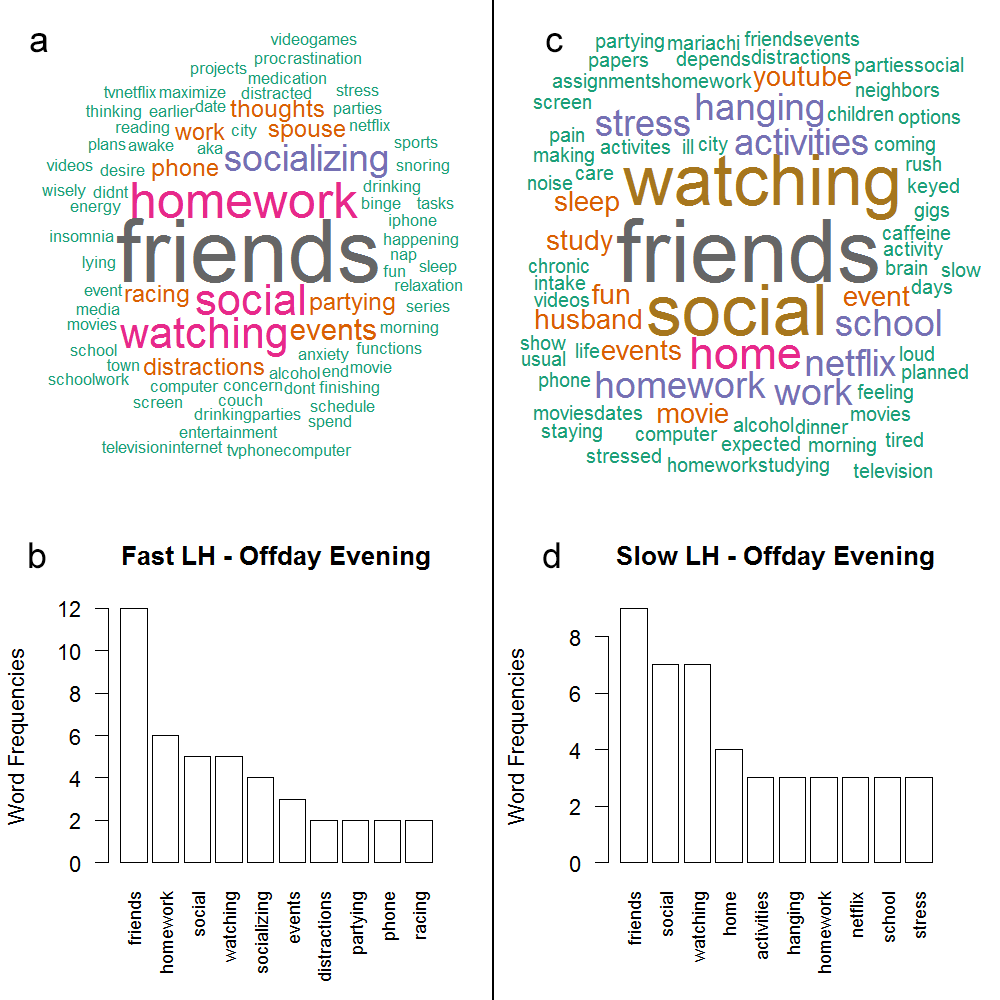


**Table S1. Study 1: Hierarchical regression results using Sleep-Wake Stability as the criterion**

A significant *b*-weight indicates the beta-weight and semi-partial correlation are also significant. *b* represents unstandardized regression weights. *beta* indicates the standardized regression weights. *sr^2^* represents the semi-partial correlation squared. *r* represents the zero-order correlation. *LL* and *UL* indicate the lower and upper limits of a confidence interval, respectively. * indicates *p* < .05. ** indicates *p* < .01.

| Predictor | *b* | *b*  95% CI  [LL, UL] | *beta* | *beta*  95% CI  [LL, UL] | *sr^2^* | *sr^2^*  95% CI  [LL, UL] | *r* | Fit | Difference |
| --- | --- | --- | --- | --- | --- | --- | --- | --- | --- |
| (Intercept) | 7.27** | [6.75, 7.79] |  |  |  |  |  |  |  |
| LH K-Factor | 0.43* | [0.06, 0.81] | 0.14 | [0.02, 0.26] | .02 | [.00, .06] | .14* |  |  |
|  |  |  |  |  |  |  |  | *R^2^*  = .019* |  |
|  |  |  |  |  |  |  |  | 95% CI[.00,.06] |  |
|  |  |  |  |  |  |  |  |  |  |
| (Intercept) | 6.90** | [4.77, 9.04] |  |  |  |  |  |  |  |
| LH K-Factor | 0.35 | [-0.05, 0.74] | 0.11 | [-0.01, 0.24] | .01 | [-.01, .04] | .14* |  |  |
| LTMO | 0.11 | [-0.18, 0.40] | 0.05 | [-0.08, 0.18] | .00 | [-.01, .01] | .09 |  |  |
| STMO | -0.07 | [-0.21, 0.08] | -0.06 | [-0.19, 0.07] | .00 | [-.01, .02] | -.11 |  |  |
|  |  |  |  |  |  |  |  | *R^2^*  = .027 | Δ*R^2^*  = .008 |
|  |  |  |  |  |  |  |  | 95% CI[.00,.07] | 95% CI[-.01, .03] |
|  |  |  |  |  |  |  |  |  |  |
| (Intercept) | 6.98** | [4.85, 9.11] |  |  |  |  |  |  |  |
| LH K-Factor | 0.42* | [0.02, 0.81] | 0.13 | [0.01, 0.26] | .02 | [-.01, .04] | .14* |  |  |
| LTMO | 0.09 | [-0.20, 0.38] | 0.04 | [-0.09, 0.17] | .00 | [-.01, .01] | .09 |  |  |
| STMO | -0.11 | [-0.25, 0.04] | -0.10 | [-0.23, 0.04] | .01 | [-.01, .03] | -.11 |  |  |
| Sex Partners | 0.00 | [-0.00, 0.01] | 0.11 | [-0.02, 0.24] | .01 | [-.01, .03] | .09 |  |  |
| Sex Partners (One-Time) | 0.02 | [-0.03, 0.07] | 0.06 | [-0.07, 0.19] | .00 | [-.01, .02] | .03 |  |  |
|  |  |  |  |  |  |  |  | *R^2^*  = .045* | Δ*R^2^*  = .018 |
|  |  |  |  |  |  |  |  | 95% CI[.00,.09] | 95% CI[-.01, .05] |
|  |  |  |  |  |  |  |  |  |  |
| (Intercept) | 4.74** | [2.42, 7.06] |  |  |  |  |  |  |  |
| LH K-Factor | 0.38 | [-0.01, 0.77] | 0.12 | [-0.00, 0.25] | .01 | [-.01, .04] | .14* |  |  |
| LTMO | 0.13 | [-0.15, 0.41] | 0.06 | [-0.07, 0.18] | .00 | [-.01, .02] | .09 |  |  |
| STMO | -0.05 | [-0.19, 0.10] | -0.04 | [-0.18, 0.09] | .00 | [-.01, .01] | -.11 |  |  |
| Sex Partners | 0.00 | [-0.00, 0.01] | 0.07 | [-0.05, 0.20] | .00 | [-.01, .02] | .09 |  |  |
| Sex Partners (One-Time) | 0.02 | [-0.03, 0.07] | 0.06 | [-0.07, 0.19] | .00 | [-.01, .01] | .03 |  |  |
| HVS-M | 0.54** | [0.29, 0.80] | 0.26 | [0.14, 0.38] | .06 | [.01, .12] | .27** |  |  |
| HVS-P | -0.01 | [-0.02, 0.00] | -0.08 | [-0.20, 0.04] | .01 | [-.01, .03] | -.04 |  |  |
|  |  |  |  |  |  |  |  | *R^2^*  = .110** | Δ*R^2^*  = .065** |
|  |  |  |  |  |  |  |  | 95% CI[.03,.16] | 95% CI[.01, .12] |
|  |  |  |  |  |  |  |  |  |  |

**Table S2. Study 1: Hierarchical regression results using Sleep Onset Latency as the criterion**

* indicates *p* < .05. ** indicates *p* < .01.

| Predictor | *b* | *b*  95% CI  [LL, UL] | *beta* | *beta*  95% CI  [LL, UL] | *sr^2^* | *sr^2^*  95% CI  [LL, UL] | *r* | Fit | Difference |
| --- | --- | --- | --- | --- | --- | --- | --- | --- | --- |
| (Intercept) | 32.34** | [26.34, 38.35] |  |  |  |  |  |  |  |
| LH K-Factor | -6.16** | [-10.48, -1.83] | -0.17 | [-0.29, -0.05] | .03 | [.00, .08] | -.17** |  |  |
|  |  |  |  |  |  |  |  | *R^2^*  = .029** |  |
|  |  |  |  |  |  |  |  | 95% CI[.00,.08] |  |
|  |  |  |  |  |  |  |  |  |  |
| (Intercept) | 40.09** | [15.47, 64.70] |  |  |  |  |  |  |  |
| LH K-Factor | -6.52** | [-11.07, -1.98] | -0.18 | [-0.31, -0.05] | .03 | [-.01, .07] | -.17** |  |  |
| LTMO | -0.73 | [-4.10, 2.65] | -0.03 | [-0.16, 0.10] | .00 | [-.01, .01] | -.05 |  |  |
| STMO | -0.74 | [-2.39, 0.92] | -0.06 | [-0.19, 0.07] | .00 | [-.01, .02] | .00 |  |  |
|  |  |  |  |  |  |  |  | *R^2^*  = .032* | Δ*R^2^*  = .003 |
|  |  |  |  |  |  |  |  | 95% CI[.00,.08] | 95% CI[-.01, .02] |
|  |  |  |  |  |  |  |  |  |  |
| (Intercept) | 40.57** | [15.80, 65.35] |  |  |  |  |  |  |  |
| LH K-Factor | -6.72** | [-11.35, -2.08] | -0.19 | [-0.32, -0.06] | .03 | [-.01, .07] | -.17** |  |  |
| LTMO | -0.76 | [-4.16, 2.63] | -0.03 | [-0.16, 0.10] | .00 | [-.01, .01] | -.05 |  |  |
| STMO | -0.64 | [-2.36, 1.09] | -0.05 | [-0.19, 0.09] | .00 | [-.01, .01] | .00 |  |  |
| Sex Partners | 0.01 | [-0.04, 0.06] | 0.02 | [-0.11, 0.15] | .00 | [-.00, .00] | .02 |  |  |
| Sex Partners (One-Time) | -0.18 | [-0.76, 0.41] | -0.04 | [-0.18, 0.09] | .00 | [-.01, .01] | -.00 |  |  |
|  |  |  |  |  |  |  |  | *R^2^*  = .034 | Δ*R^2^*  = .001 |
|  |  |  |  |  |  |  |  | 95% CI[.00,.07] | 95% CI[-.01, .01] |
|  |  |  |  |  |  |  |  |  |  |
| (Intercept) | 51.03** | [23.25, 78.81] |  |  |  |  |  |  |  |
| LH K-Factor | -6.55** | [-11.19, -1.92] | -0.18 | [-0.31, -0.05] | .03 | [-.01, .07] | -.17** |  |  |
| LTMO | -0.94 | [-4.34, 2.46] | -0.04 | [-0.17, 0.09] | .00 | [-.01, .01] | -.05 |  |  |
| STMO | -0.91 | [-2.65, 0.84] | -0.07 | [-0.21, 0.07] | .00 | [-.01, .02] | .00 |  |  |
| Sex Partners | 0.01 | [-0.04, 0.06] | 0.03 | [-0.10, 0.16] | .00 | [-.01, .01] | .02 |  |  |
| Sex Partners (One-Time) | -0.17 | [-0.75, 0.41] | -0.04 | [-0.17, 0.10] | .00 | [-.01, .01] | -.00 |  |  |
| HVS-M | -2.53 | [-5.54, 0.47] | -0.11 | [-0.23, 0.02] | .01 | [-.01, .03] | -.10 |  |  |
| HVS-P | 0.04 | [-0.11, 0.18] | 0.03 | [-0.09, 0.15] | .00 | [-.01, .01] | .01 |  |  |
|  |  |  |  |  |  |  |  | *R^2^*  = .044 | Δ*R^2^*  = .010 |
|  |  |  |  |  |  |  |  | 95% CI[.00,.08] | 95% CI[-.01, .03] |
|  |  |  |  |  |  |  |  |  |  |

**Table S3. Study 1: Hierarchical regression results using Wakefulness After Sleep Onset as the criterion**

* indicates *p* < .05. ** indicates *p* < .01.

| Predictor | *b* | *b*  95% CI  [LL, UL] | *beta* | *beta*  95% CI  [LL, UL] | *sr^2^* | *sr^2^*  95% CI  [LL, UL] | *r* | Fit | Difference |
| --- | --- | --- | --- | --- | --- | --- | --- | --- | --- |
| (Intercept) | 29.09** | [22.00, 36.18] |  |  |  |  |  |  |  |
| LH K-Factor | -6.35* | [-11.45, -1.24] | -0.15 | [-0.27, -0.03] | .02 | [.00, .07] | -.15* |  |  |
|  |  |  |  |  |  |  |  | *R^2^*  = .022* |  |
|  |  |  |  |  |  |  |  | 95% CI[.00,.07] |  |
|  |  |  |  |  |  |  |  |  |  |
| (Intercept) | 16.82 | [-12.16, 45.81] |  |  |  |  |  |  |  |
| LH K-Factor | -7.52** | [-12.87, -2.17] | -0.18 | [-0.30, -0.05] | .03 | [-.01, .07] | -.15* |  |  |
| LTMO | 2.45 | [-1.52, 6.43] | 0.08 | [-0.05, 0.21] | .01 | [-.01, .02] | .06 |  |  |
| STMO | -0.60 | [-2.55, 1.35] | -0.04 | [-0.17, 0.09] | .00 | [-.01, .01] | -.02 |  |  |
|  |  |  |  |  |  |  |  | *R^2^*  = .032* | Δ*R^2^*  = .009 |
|  |  |  |  |  |  |  |  | 95% CI[.00,.07] | 95% CI[-.01, .03] |
|  |  |  |  |  |  |  |  |  |  |
| (Intercept) | 17.16 | [-11.37, 45.69] |  |  |  |  |  |  |  |
| LH K-Factor | -5.87* | [-11.21, -0.53] | -0.14 | [-0.26, -0.01] | .02 | [-.01, .05] | -.15* |  |  |
| LTMO | 2.19 | [-1.73, 6.10] | 0.07 | [-0.06, 0.20] | .00 | [-.01, .02] | .06 |  |  |
| STMO | -1.51 | [-3.50, 0.47] | -0.10 | [-0.24, 0.03] | .01 | [-.01, .03] | -.02 |  |  |
| Sex Partners | 0.05 | [-0.00, 0.11] | 0.12 | [-0.01, 0.24] | .01 | [-.01, .04] | .17** |  |  |
| Sex Partners (One-Time) | 0.80* | [0.13, 1.47] | 0.16 | [0.02, 0.29] | .02 | [-.01, .05] | .19** |  |  |
|  |  |  |  |  |  |  |  | *R^2^*  = .075** | Δ*R^2^*  = .043** |
|  |  |  |  |  |  |  |  | 95% CI[.01,.13] | 95% CI[-.00, .09] |
|  |  |  |  |  |  |  |  |  |  |
| (Intercept) | 21.30 | [-10.69, 53.29] |  |  |  |  |  |  |  |
| LH K-Factor | -5.75* | [-11.08, -0.41] | -0.14 | [-0.26, -0.01] | .02 | [-.01, .05] | -.15* |  |  |
| LTMO | 2.28 | [-1.64, 6.20] | 0.07 | [-0.05, 0.20] | .00 | [-.01, .02] | .06 |  |  |
| STMO | -1.56 | [-3.57, 0.46] | -0.10 | [-0.24, 0.03] | .01 | [-.01, .03] | -.02 |  |  |
| Sex Partners | 0.05 | [-0.00, 0.11] | 0.12 | [-0.01, 0.24] | .01 | [-.01, .04] | .17** |  |  |
| Sex Partners (One-Time) | 0.80* | [0.13, 1.47] | 0.16 | [0.03, 0.29] | .02 | [-.01, .05] | .19** |  |  |
| HVS-M | -0.68 | [-4.14, 2.78] | -0.02 | [-0.15, 0.10] | .00 | [-.00, .01] | -.02 |  |  |
| HVS-P | -0.13 | [-0.30, 0.04] | -0.09 | [-0.21, 0.03] | .01 | [-.01, .03] | -.10 |  |  |
|  |  |  |  |  |  |  |  | *R^2^*  = .085** | Δ*R^2^*  = .010 |
|  |  |  |  |  |  |  |  | 95% CI[.01,.13] | 95% CI[-.01, .03] |
|  |  |  |  |  |  |  |  |  |  |

**Table S4. Study 1: Hierarchical regression results using Hypothetical Sleep Duration Variability as the criterion**

* indicates *p* < .05. ** indicates *p* < .01.

| Predictor | *b* | *b*  95% CI  [LL, UL] | *beta* | *beta*  95% CI  [LL, UL] | *sr^2^* | *sr^2^*  95% CI  [LL, UL] | *r* | Fit | Difference |
| --- | --- | --- | --- | --- | --- | --- | --- | --- | --- |
| (Intercept) | 327.61** | [296.70, 358.51] |  |  |  |  |  |  |  |
| LH K-Factor | -29.40** | [-51.66, -7.14] | -0.16 | [-0.28, -0.04] | .03 | [.00, .07] | -.16** |  |  |
|  |  |  |  |  |  |  |  | *R^2^*  = .025** |  |
|  |  |  |  |  |  |  |  | 95% CI[.00,.07] |  |
|  |  |  |  |  |  |  |  |  |  |
| (Intercept) | 237.21** | [111.51, 362.91] |  |  |  |  |  |  |  |
| LH K-Factor | -23.98* | [-47.19, -0.78] | -0.13 | [-0.26, -0.00] | .02 | [-.01, .04] | -.16** |  |  |
| LTMO | 7.66 | [-9.58, 24.90] | 0.06 | [-0.07, 0.18] | .00 | [-.01, .02] | -.02 |  |  |
| STMO | 9.71* | [1.27, 18.16] | 0.15 | [0.02, 0.28] | .02 | [-.01, .05] | .17** |  |  |
|  |  |  |  |  |  |  |  | *R^2^*  = .044** | Δ*R^2^*  = .019 |
|  |  |  |  |  |  |  |  | 95% CI[.00,.09] | 95% CI[-.01, .05] |
|  |  |  |  |  |  |  |  |  |  |
| (Intercept) | 225.19** | [101.25, 349.13] |  |  |  |  |  |  |  |
| LH K-Factor | -27.98* | [-51.18, -4.79] | -0.15 | [-0.28, -0.03] | .02 | [-.01, .05] | -.16** |  |  |
| LTMO | 9.77 | [-7.23, 26.77] | 0.07 | [-0.05, 0.20] | .00 | [-.01, .02] | -.02 |  |  |
| STMO | 12.08** | [3.46, 20.70] | 0.19 | [0.05, 0.32] | .03 | [-.01, .06] | .17** |  |  |
| Sex Partners | -0.40** | [-0.65, -0.15] | -0.20 | [-0.33, -0.08] | .04 | [-.01, .08] | -.15* |  |  |
| Sex Partners (One-Time) | -0.15 | [-3.06, 2.76] | -0.01 | [-0.14, 0.12] | .00 | [-.00, .00] | .02 |  |  |
|  |  |  |  |  |  |  |  | *R^2^*  = .084** | Δ*R^2^*  = .040** |
|  |  |  |  |  |  |  |  | 95% CI[.02,.14] | 95% CI[-.01, .08] |
|  |  |  |  |  |  |  |  |  |  |
| (Intercept) | 345.15** | [210.25, 480.04] |  |  |  |  |  |  |  |
| LH K-Factor | -26.20* | [-48.71, -3.69] | -0.14 | [-0.26, -0.02] | .02 | [-.01, .05] | -.16** |  |  |
| LTMO | 7.31 | [-9.20, 23.83] | 0.05 | [-0.07, 0.18] | .00 | [-.01, .01] | -.02 |  |  |
| STMO | 8.81* | [0.32, 17.30] | 0.14 | [0.00, 0.27] | .01 | [-.01, .04] | .17** |  |  |
| Sex Partners | -0.34** | [-0.58, -0.10] | -0.17 | [-0.29, -0.05] | .03 | [-.01, .06] | -.15* |  |  |
| Sex Partners (One-Time) | -0.05 | [-2.87, 2.77] | -0.00 | [-0.13, 0.12] | .00 | [-.00, .00] | .02 |  |  |
| HVS-M | -29.91** | [-44.50, -15.32] | -0.24 | [-0.36, -0.12] | .05 | [.00, .11] | -.27** |  |  |
| HVS-P | 0.79* | [0.08, 1.51] | 0.13 | [0.01, 0.25] | .02 | [-.01, .04] | .09 |  |  |
|  |  |  |  |  |  |  |  | *R^2^*  = .146** | Δ*R^2^*  = .062** |
|  |  |  |  |  |  |  |  | 95% CI[.06,.21] | 95% CI[.01, .12] |
|  |  |  |  |  |  |  |  |  |  |

**Table S5. Study 1: Hierarchical regression results using Sleep Duration Variability as the criterion**

* indicates *p* < .05. ** indicates *p* < .01.

| Predictor | *b* | *b*  95% CI  [LL, UL] | *beta* | *beta*  95% CI  [LL, UL] | *sr^2^* | *sr^2^*  95% CI  [LL, UL] | *r* | Fit | Difference |
| --- | --- | --- | --- | --- | --- | --- | --- | --- | --- |
| (Intercept) | 91.29** | [80.51, 102.07] |  |  |  |  |  |  |  |
| LH K-Factor | -6.59 | [-14.35, 1.18] | -0.10 | [-0.22, 0.02] | .01 | [.00, .05] | -.10 |  |  |
|  |  |  |  |  |  |  |  | *R^2^*  = .011 |  |
|  |  |  |  |  |  |  |  | 95% CI[.00,.05] |  |
|  |  |  |  |  |  |  |  |  |  |
| (Intercept) | 66.97** | [22.80, 111.15] |  |  |  |  |  |  |  |
| LH K-Factor | -6.57 | [-14.73, 1.58] | -0.10 | [-0.23, 0.02] | .01 | [-.01, .03] | -.10 |  |  |
| LTMO | 3.13 | [-2.93, 9.19] | 0.07 | [-0.06, 0.20] | .00 | [-.01, .02] | .03 |  |  |
| STMO | 1.16 | [-1.80, 4.13] | 0.05 | [-0.08, 0.18] | .00 | [-.01, .01] | .06 |  |  |
|  |  |  |  |  |  |  |  | *R^2^*  = .015 | Δ*R^2^*  = .005 |
|  |  |  |  |  |  |  |  | 95% CI[.00,.05] | 95% CI[-.01, .02] |
|  |  |  |  |  |  |  |  |  |  |
| (Intercept) | 68.84** | [24.49, 113.19] |  |  |  |  |  |  |  |
| LH K-Factor | -6.12 | [-14.42, 2.18] | -0.10 | [-0.23, 0.03] | .01 | [-.01, .03] | -.10 |  |  |
| LTMO | 2.82 | [-3.26, 8.91] | 0.06 | [-0.07, 0.19] | .00 | [-.01, .02] | .03 |  |  |
| STMO | 0.89 | [-2.19, 3.97] | 0.04 | [-0.10, 0.18] | .00 | [-.01, .01] | .06 |  |  |
| Sex Partners | 0.06 | [-0.03, 0.15] | 0.08 | [-0.05, 0.21] | .01 | [-.01, .03] | .10 |  |  |
| Sex Partners (One-Time) | -0.06 | [-1.11, 0.98] | -0.01 | [-0.14, 0.13] | .00 | [-.00, .00] | .05 |  |  |
|  |  |  |  |  |  |  |  | *R^2^*  = .022 | Δ*R^2^*  = .006 |
|  |  |  |  |  |  |  |  | 95% CI[.00,.05] | 95% CI[-.01, .03] |
|  |  |  |  |  |  |  |  |  |  |
| (Intercept) | 97.78** | [48.40, 147.15] |  |  |  |  |  |  |  |
| LH K-Factor | -5.64 | [-13.87, 2.60] | -0.09 | [-0.22, 0.04] | .01 | [-.01, .03] | -.10 |  |  |
| LTMO | 2.39 | [-3.65, 8.44] | 0.05 | [-0.08, 0.18] | .00 | [-.01, .01] | .03 |  |  |
| STMO | 0.16 | [-2.94, 3.27] | 0.01 | [-0.13, 0.15] | .00 | [-.00, .00] | .06 |  |  |
| Sex Partners | 0.07 | [-0.02, 0.16] | 0.10 | [-0.02, 0.23] | .01 | [-.01, .03] | .10 |  |  |
| Sex Partners (One-Time) | -0.04 | [-1.07, 0.99] | -0.01 | [-0.14, 0.13] | .00 | [-.00, .00] | .05 |  |  |
| HVS-M | -6.90* | [-12.24, -1.56] | -0.16 | [-0.29, -0.04] | .02 | [-.01, .06] | -.15* |  |  |
| HVS-P | 0.05 | [-0.21, 0.31] | 0.02 | [-0.10, 0.14] | .00 | [-.00, .01] | -.00 |  |  |
|  |  |  |  |  |  |  |  | *R^2^*  = .046 | Δ*R^2^*  = .024* |
|  |  |  |  |  |  |  |  | 95% CI[.00,.08] | 95% CI[-.01, .06] |
|  |  |  |  |  |  |  |  |  |  |

**Table S6. Study 1: Hierarchical regression results using Eveningness as the criterion**

* indicates *p* < .05. ** indicates *p* < .01.

| Predictor | *b* | *b*  95% CI  [LL, UL] | *beta* | *beta*  95% CI  [LL, UL] | *sr^2^* | *sr^2^*  95% CI  [LL, UL] | *r* | Fit | Difference |
| --- | --- | --- | --- | --- | --- | --- | --- | --- | --- |
| (Intercept) | 8.17** | [7.58, 8.77] |  |  |  |  |  |  |  |
| LH K-Factor | -0.26 | [-0.69, 0.17] | -0.07 | [-0.20, 0.05] | .01 | [.00, .04] | -.07 |  |  |
|  |  |  |  |  |  |  |  | *R^2^*  = .005 |  |
|  |  |  |  |  |  |  |  | 95% CI[.00,.04] |  |
|  |  |  |  |  |  |  |  |  |  |
| (Intercept) | 6.04** | [3.63, 8.45] |  |  |  |  |  |  |  |
| LH K-Factor | -0.11 | [-0.55, 0.33] | -0.03 | [-0.16, 0.09] | .00 | [-.01, .01] | -.07 |  |  |
| LTMO | 0.16 | [-0.17, 0.49] | 0.06 | [-0.07, 0.19] | .00 | [-.01, .02] | -.01 |  |  |
| STMO | 0.25** | [0.09, 0.42] | 0.20 | [0.07, 0.33] | .04 | [-.01, .08] | .19** |  |  |
|  |  |  |  |  |  |  |  | *R^2^*  = .041* | Δ*R^2^*  = .035** |
|  |  |  |  |  |  |  |  | 95% CI[.00,.09] | 95% CI[-.01, .08] |
|  |  |  |  |  |  |  |  |  |  |
| (Intercept) | 6.03** | [3.66, 8.39] |  |  |  |  |  |  |  |
| LH K-Factor | -0.26 | [-0.70, 0.19] | -0.07 | [-0.20, 0.05] | .00 | [-.01, .02] | -.07 |  |  |
| LTMO | 0.18 | [-0.14, 0.51] | 0.07 | [-0.05, 0.20] | .00 | [-.01, .02] | -.01 |  |  |
| STMO | 0.33** | [0.17, 0.50] | 0.27 | [0.14, 0.40] | .06 | [.00, .11] | .19** |  |  |
| Sex Partners | -0.00 | [-0.01, 0.00] | -0.11 | [-0.24, 0.01] | .01 | [-.01, .04] | -.11 |  |  |
| Sex Partners (One-Time) | -0.07* | [-0.13, -0.02] | -0.17 | [-0.30, -0.04] | .02 | [-.01, .06] | -.11 |  |  |
|  |  |  |  |  |  |  |  | *R^2^*  = .089** | Δ*R^2^*  = .048** |
|  |  |  |  |  |  |  |  | 95% CI[.02,.14] | 95% CI[-.00, .10] |
|  |  |  |  |  |  |  |  |  |  |
| (Intercept) | 7.10** | [4.49, 9.71] |  |  |  |  |  |  |  |
| LH K-Factor | -0.25 | [-0.68, 0.19] | -0.07 | [-0.19, 0.05] | .00 | [-.01, .02] | -.07 |  |  |
| LTMO | 0.15 | [-0.17, 0.47] | 0.06 | [-0.07, 0.18] | .00 | [-.01, .01] | -.01 |  |  |
| STMO | 0.30** | [0.13, 0.46] | 0.24 | [0.11, 0.37] | .04 | [-.00, .09] | .19** |  |  |
| Sex Partners | -0.00 | [-0.01, 0.00] | -0.10 | [-0.22, 0.03] | .01 | [-.01, .03] | -.11 |  |  |
| Sex Partners (One-Time) | -0.07** | [-0.13, -0.02] | -0.17 | [-0.30, -0.04] | .02 | [-.01, .06] | -.11 |  |  |
| HVS-M | -0.30* | [-0.58, -0.01] | -0.13 | [-0.25, -0.01] | .01 | [-.01, .04] | -.15* |  |  |
| HVS-P | 0.02** | [0.01, 0.03] | 0.18 | [0.06, 0.29] | .03 | [-.01, .07] | .16* |  |  |
|  |  |  |  |  |  |  |  | *R^2^*  = .127** | Δ*R^2^*  = .039** |
|  |  |  |  |  |  |  |  | 95% CI[.04,.18] | 95% CI[-.00, .08] |
|  |  |  |  |  |  |  |  |  |  |

**Figure S3. Study 2: Self-Reported Sleep Detriments for a Weekday Evening**

Word clouds were built from a free-response item asking participants about the biggest factor that delayed their bedtime. Larger words in word clouds indicate higher frequencies of word usage. Fast and Slow LH refer to the lowest and highest tertile of the sample LH K-Factor distribution as measured by the Mini-K scale. (a) Word cloud of Fast-LH participants’ responses (b) The ten most frequently used words in Fast-LH responses (c) Word cloud of Slow-LH participants’ responses (d) The ten most frequently used words in Slow-LH responses


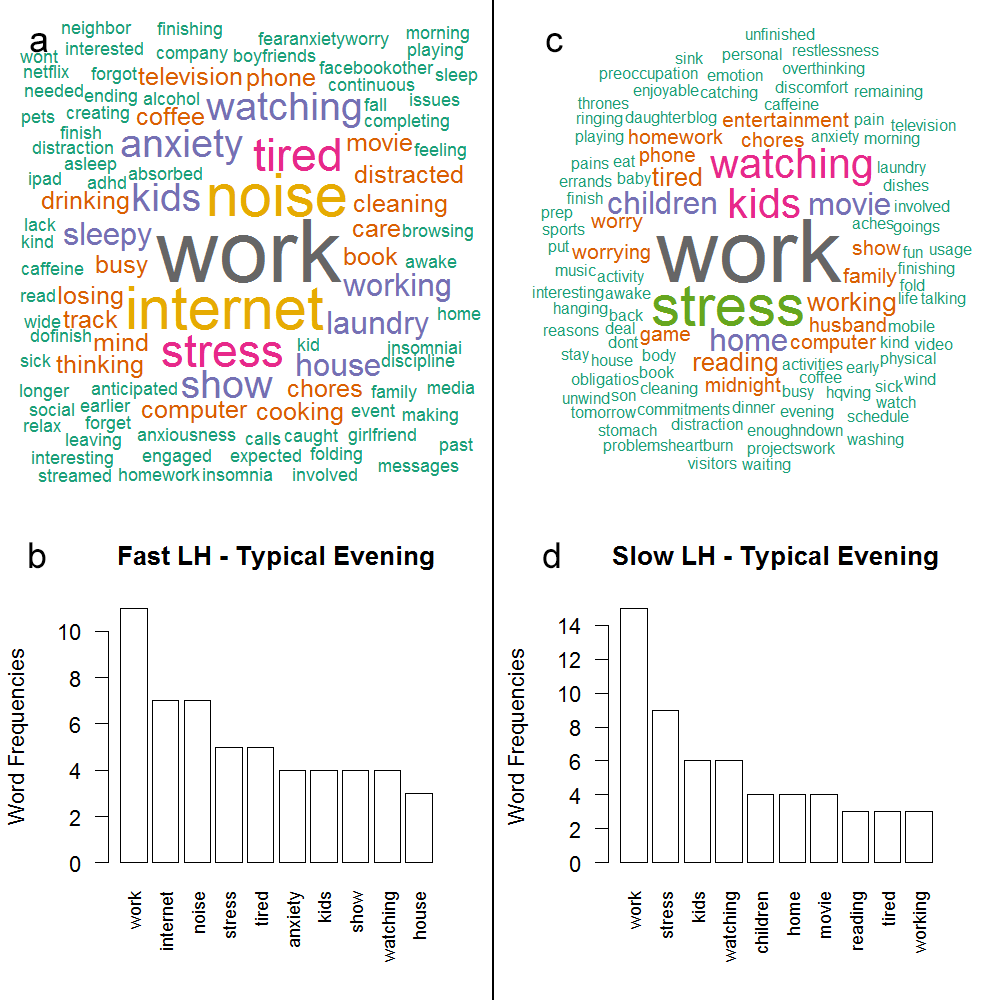


**Figure S4. Study 2: Self-Reported Sleep Detriments for a Weekend Evening**

Word clouds were built from a free-response item asking participants about the biggest factor that delayed their bedtime. Larger words in word clouds indicate higher frequencies of word usage. Fast and Slow LH refer to the lowest and highest tertile of the sample LH K-Factor distribution as measured by the Mini-K scale. (a) Word cloud of Fast-LH participants’ responses (b) The ten most frequently used words in Fast-LH responses (c) Word cloud of Slow-LH participants’ responses (d) The ten most frequently used words in Slow-LH responses


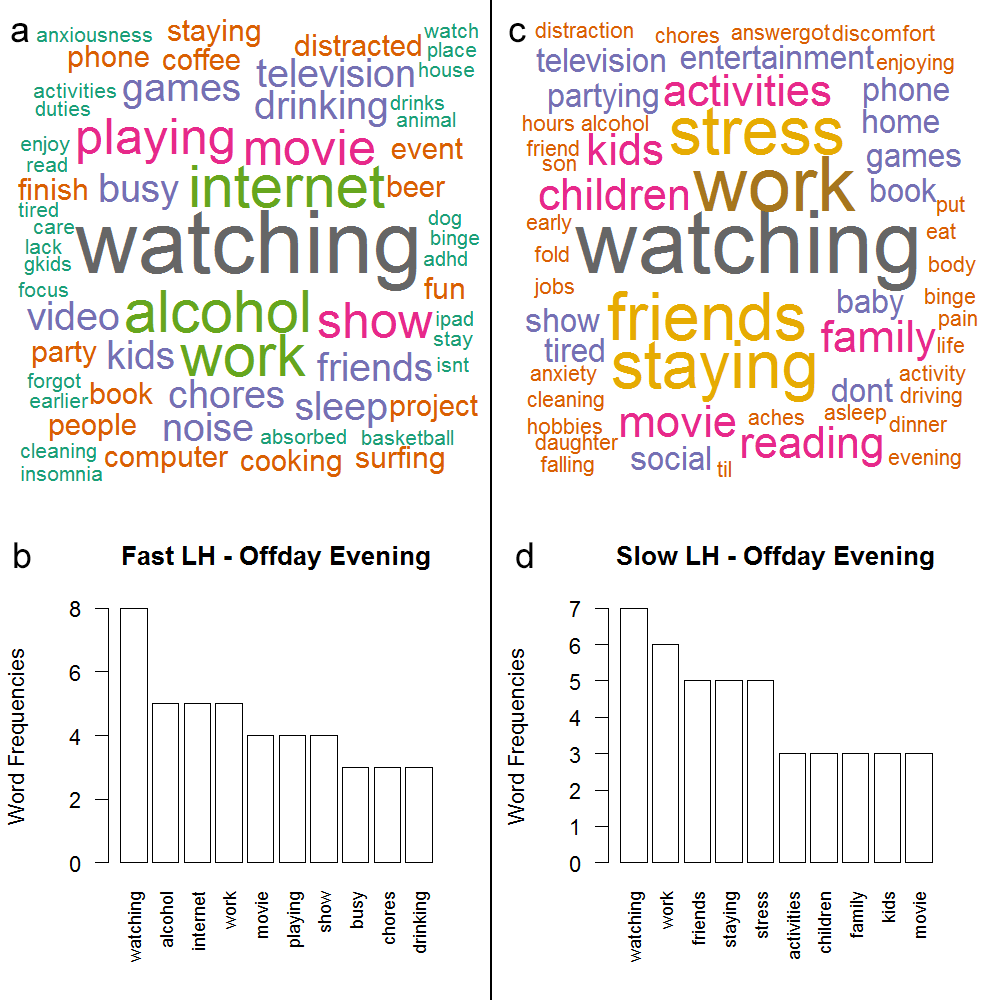


**Figure S5. Study 2: Life History Strategy Versus Habitual Sleep Duration (Full Sample)**

Values which are *lower* on the y-axis represent *slower* LH strategies (higher K-Factor). Shaded regions represent the 95% confidence interval. (A) LOESS (Locally Estimated Scatterplot Smoothing) model (span = .75). (B) Segmented regression model; the dotted vertical line indicates the estimated breakpoint (6.85 hr). Compared to the null model, the breakpoint model did not achieve a significantly better fit (*p* = .196). Ages range from 19 to 73 (M = 38.72 years, SD = 12.14). N=305.


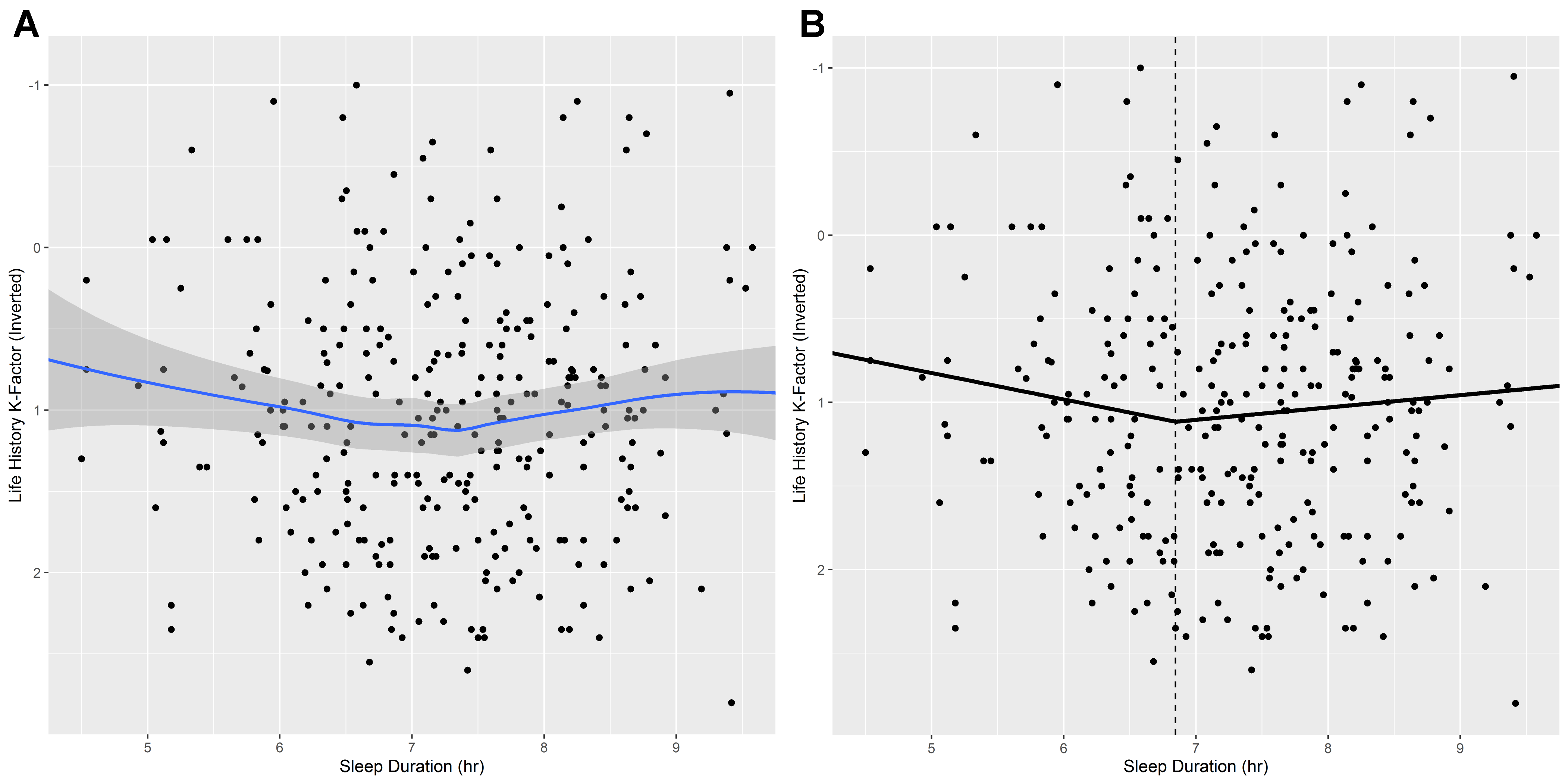


**Table S7. Study 2: Life History, Sociosexual Orientation, and Sleep-related Variables: Descriptive Statistics and Correlations with Confidence Intervals**

*M* and *SD* are used to represent mean and standard deviation, respectively. Values in square brackets indicate the 95% confidence interval for each correlation. * indicates *p* < .05. ** indicates *p* < .01.

| Variable | *M* | *SD* | 1 | 2 | 3 | 4 | 5 | 6 | 7 | 8 | 9 | 10 | 11 |
| --- | --- | --- | --- | --- | --- | --- | --- | --- | --- | --- | --- | --- | --- |
|  |  |  |  |  |  |  |  |  |  |  |  |  |  |
| 1. LH K-Factor | 1.02 | 0.79 |  |  |  |  |  |  |  |  |  |  |  |
|  |  |  |  |  |  |  |  |  |  |  |  |  |  |
| 2. STMO | 3.36 | 2.05 | -.19** |  |  |  |  |  |  |  |  |  |  |
|  |  |  | [-.30, -.08] |  |  |  |  |  |  |  |  |  |  |
|  |  |  |  |  |  |  |  |  |  |  |  |  |  |
| 3. LTMO | 6.17 | 1.28 | .35** | -.20** |  |  |  |  |  |  |  |  |  |
|  |  |  | [.25, .45] | [-.30, -.09] |  |  |  |  |  |  |  |  |  |
|  |  |  |  |  |  |  |  |  |  |  |  |  |  |
| 4. Sex Partners | 13.07 | 28.97 | -.07 | .27** | -.10 |  |  |  |  |  |  |  |  |
|  |  |  | [-.18, .04] | [.16, .37] | [-.21, .01] |  |  |  |  |  |  |  |  |
|  |  |  |  |  |  |  |  |  |  |  |  |  |  |
| 5. Eveningness | 6.77 | 2.66 | -.11* | .09 | .05 | -.19** |  |  |  |  |  |  |  |
|  |  |  | [-.22, -.00] | [-.03, .20] | [-.06, .16] | [-.30, -.08] |  |  |  |  |  |  |  |
|  |  |  |  |  |  |  |  |  |  |  |  |  |  |
| 6. HVS-M | 3.74 | 1.13 | .13* | -.16** | .07 | -.04 | -.19** |  |  |  |  |  |  |
|  |  |  | [.02, .24] | [-.27, -.05] | [-.04, .18] | [-.15, .07] | [-.30, -.08] |  |  |  |  |  |  |
|  |  |  |  |  |  |  |  |  |  |  |  |  |  |
| 7. SWS | 8.30 | 1.83 | .13* | -.14* | .10 | .04 | -.34** | .26** |  |  |  |  |  |
|  |  |  | [.02, .24] | [-.25, -.03] | [-.01, .21] | [-.07, .15] | [-.43, -.23] | [.16, .37] |  |  |  |  |  |
|  |  |  |  |  |  |  |  |  |  |  |  |  |  |
| 8. SDV | 69.93 | 49.53 | -.17** | .15* | -.10 | -.02 | .14* | -.07 | -.18** |  |  |  |  |
|  |  |  | [-.27, -.05] | [.03, .25] | [-.21, .01] | [-.14, .09] | [.03, .25] | [-.18, .04] | [-.28, -.07] |  |  |  |  |
|  |  |  |  |  |  |  |  |  |  |  |  |  |  |
| 9. H-SDV | 231.61 | 119.69 | -.16** | .24** | -.04 | -.05 | .47** | -.18** | -.39** | .46** |  |  |  |
|  |  |  | [-.26, -.04] | [.13, .34] | [-.15, .08] | [-.16, .06] | [.37, .55] | [-.29, -.07] | [-.48, -.29] | [.37, .55] |  |  |  |
|  |  |  |  |  |  |  |  |  |  |  |  |  |  |
| 10. SOL | 26.13 | 21.30 | -.19** | -.02 | -.12* | -.01 | .17** | -.12* | -.28** | .12* | .18** |  |  |
|  |  |  | [-.29, -.08] | [-.14, .09] | [-.23, -.01] | [-.12, .11] | [.06, .28] | [-.23, -.01] | [-.38, -.18] | [.00, .23] | [.07, .29] |  |  |
|  |  |  |  |  |  |  |  |  |  |  |  |  |  |
| 11. WASO | 25.83 | 30.09 | -.06 | -.04 | -.12* | .06 | .00 | .03 | -.13* | .06 | .07 | .55** |  |
|  |  |  | [-.17, .06] | [-.16, .07] | [-.23, -.01] | [-.05, .17] | [-.11, .11] | [-.08, .14] | [-.24, -.02] | [-.05, .17] | [-.04, .18] | [.47, .63] |  |
|  |  |  |  |  |  |  |  |  |  |  |  |  |  |
| 12. Sleepiness | 4.31 | 1.74 | -.24** | -.03 | .03 | .08 | -.06 | -.12* | -.04 | .04 | .02 | .15** | .17** |
|  |  |  | [-.34, -.13] | [-.14, .08] | [-.08, .14] | [-.03, .19] | [-.17, .05] | [-.23, -.01] | [-.15, .07] | [-.08, .15] | [-.09, .14] | [.04, .26] | [.06, .28] |
|  |  |  |  |  |  |  |  |  |  |  |  |  |  |

**Table S8. Study 2: Hierarchical regression results using Sleepiness as the criterion**

* indicates *p* < .05. ** indicates *p* < .01. Gender was coded as female = 0, male = 1. Income was z-scored to provide an interpretable unstandardized coefficient (*b*).

| Predictor | *b* | *b*  95% CI  [LL, UL] | *beta* | *beta*  95% CI  [LL, UL] | *sr^2^* | *sr^2^*  95% CI  [LL, UL] | *r* | Fit | Difference |
| --- | --- | --- | --- | --- | --- | --- | --- | --- | --- |
| (Intercept) | 4.84** | [4.53, 5.15] |  |  |  |  |  |  |  |
| LH K-Factor | -0.52** | [-0.76, -0.28] | -0.24 | [-0.34, -0.13] | .06 | [.02, .11] | -.24** |  |  |
|  |  |  |  |  |  |  |  | *R^2^*  = .055** |  |
|  |  |  |  |  |  |  |  | 95% CI[.02,.11] |  |
|  |  |  |  |  |  |  |  |  |  |
| (Intercept) | 4.14** | [3.07, 5.20] |  |  |  |  |  |  |  |
| LH K-Factor | -0.64** | [-0.90, -0.38] | -0.29 | [-0.41, -0.17] | .07 | [.02, .13] | -.24** |  |  |
| LTMO | 0.16* | [0.00, 0.32] | 0.12 | [0.00, 0.24] | .01 | [-.01, .04] | .03 |  |  |
| STMO | -0.05 | [-0.15, 0.04] | -0.06 | [-0.18, 0.05] | .00 | [-.01, .02] | -.03 |  |  |
|  |  |  |  |  |  |  |  | *R^2^*  = .074** | Δ*R^2^*  = .018 |
|  |  |  |  |  |  |  |  | 95% CI[.02,.13] | 95% CI[-.01, .05] |
|  |  |  |  |  |  |  |  |  |  |
| (Intercept) | 4.11** | [3.04, 5.18] |  |  |  |  |  |  |  |
| LH K-Factor | -0.64** | [-0.90, -0.38] | -0.29 | [-0.41, -0.17] | .07 | [.02, .13] | -.24** |  |  |
| LTMO | 0.16* | [0.00, 0.33] | 0.12 | [0.00, 0.24] | .01 | [-.01, .04] | .03 |  |  |
| STMO | -0.08 | [-0.18, 0.02] | -0.09 | [-0.21, 0.03] | .01 | [-.01, .03] | -.03 |  |  |
| Sex Partners | 0.01 | [-0.01, 0.03] | 0.15 | [-0.15, 0.46] | .00 | [-.01, .01] | .08 |  |  |
| Sex Partners (One-Time) | -0.00 | [-0.03, 0.02] | -0.06 | [-0.36, 0.25] | .00 | [-.00, .00] | .08 |  |  |
|  |  |  |  |  |  |  |  | *R^2^*  = .083** | Δ*R^2^*  = .010 |
|  |  |  |  |  |  |  |  | 95% CI[.02,.13] | 95% CI[-.01, .03] |
|  |  |  |  |  |  |  |  |  |  |
| (Intercept) | 4.73** | [3.48, 5.97] |  |  |  |  |  |  |  |
| LH K-Factor | -0.61** | [-0.87, -0.35] | -0.28 | [-0.39, -0.16] | .06 | [.01, .12] | -.24** |  |  |
| LTMO | 0.17* | [0.01, 0.33] | 0.12 | [0.00, 0.24] | .01 | [-.01, .04] | .03 |  |  |
| STMO | -0.09 | [-0.19, 0.01] | -0.11 | [-0.22, 0.01] | .01 | [-.01, .03] | -.03 |  |  |
| Sex Partners | 0.01 | [-0.01, 0.03] | 0.13 | [-0.17, 0.44] | .00 | [-.01, .01] | .08 |  |  |
| Sex Partners (One-Time) | -0.00 | [-0.03, 0.02] | -0.03 | [-0.34, 0.27] | .00 | [-.00, .00] | .08 |  |  |
| HVS-M | -0.18* | [-0.35, -0.00] | -0.11 | [-0.23, -0.00] | .01 | [-.01, .04] | -.12* |  |  |
| HVS-P | 0.00 | [-0.01, 0.01] | 0.03 | [-0.08, 0.14] | .00 | [-.01, .01] | .01 |  |  |
|  |  |  |  |  |  |  |  | *R^2^*  = .095** | Δ*R^2^*  = .012 |
|  |  |  |  |  |  |  |  | 95% CI[.03,.14] | 95% CI[-.01, .04] |
|  |  |  |  |  |  |  |  |  |  |
| (Intercept) | 6.32** | [4.82, 7.82] |  |  |  |  |  |  |  |
| LH K-Factor | -0.56** | [-0.82, -0.30] | -0.25 | [-0.37, -0.13] | .05 | [.01, .10] | -.24** |  |  |
| LTMO | 0.15 | [-0.01, 0.31] | 0.11 | [-0.00, 0.23] | .01 | [-.01, .03] | .03 |  |  |
| STMO | -0.02 | [-0.13, 0.09] | -0.02 | [-0.15, 0.11] | .00 | [-.00, .00] | -.03 |  |  |
| Sex Partners | 0.00 | [-0.01, 0.02] | 0.06 | [-0.25, 0.37] | .00 | [-.00, .00] | .08 |  |  |
| Sex Partners (One-Time) | 0.00 | [-0.02, 0.03] | 0.05 | [-0.25, 0.36] | .00 | [-.00, .00] | .08 |  |  |
| HVS-M | -0.19* | [-0.37, -0.02] | -0.13 | [-0.24, -0.01] | .01 | [-.01, .04] | -.12* |  |  |
| HVS-P | -0.00 | [-0.01, 0.01] | -0.01 | [-0.12, 0.10] | .00 | [-.00, .00] | .01 |  |  |
| Gender | -0.86** | [-1.30, -0.42] | -0.25 | [-0.37, -0.12] | .04 | [.00, .08] | -.18** |  |  |
| Age | -0.02** | [-0.04, -0.01] | -0.15 | [-0.27, -0.04] | .02 | [-.01, .05] | -.13* |  |  |
| Income | 0.13 | [-0.07, 0.33] | 0.07 | [-0.04, 0.19] | .00 | [-.01, .02] | -.05 |  |  |
| Education | -0.09 | [-0.21, 0.03] | -0.08 | [-0.19, 0.03] | .01 | [-.01, .02] | -.09 |  |  |
| Parenthood | -0.35 | [-0.76, 0.05] | -0.10 | [-0.21, 0.01] | .01 | [-.01, .03] | -.07 |  |  |
|  |  |  |  |  |  |  |  | *R^2^*  = .163** | Δ*R^2^*  = .067** |
|  |  |  |  |  |  |  |  | 95% CI[.06,.21] | 95% CI[.02, .12] |
|  |  |  |  |  |  |  |  |  |  |

**Table S9. Study 2: Hierarchical regression results using Sleep Onset Latency as the criterion**

* indicates *p* < .05. ** indicates *p* < .01. Gender was coded as female = 0, male = 1. Income was z-scored to provide an interpretable unstandardized coefficient (*b*).

| Predictor | *b* | *b*  95% CI  [LL, UL] | *beta* | *beta*  95% CI  [LL, UL] | *sr^2^* | *sr^2^*  95% CI  [LL, UL] | *r* | Fit | Difference |
| --- | --- | --- | --- | --- | --- | --- | --- | --- | --- |
| (Intercept) | 31.26** | [27.40, 35.13] |  |  |  |  |  |  |  |
| LH K-Factor | -5.02** | [-8.02, -2.03] | -0.19 | [-0.30, -0.08] | .03 | [.01, .08] | -.19** |  |  |
|  |  |  |  |  |  |  |  | *R^2^*  = .035** |  |
|  |  |  |  |  |  |  |  | 95% CI[.01,.08] |  |
|  |  |  |  |  |  |  |  |  |  |
| (Intercept) | 40.94** | [27.70, 54.19] |  |  |  |  |  |  |  |
| LH K-Factor | -4.70** | [-7.94, -1.47] | -0.17 | [-0.29, -0.05] | .03 | [-.01, .06] | -.19** |  |  |
| LTMO | -1.21 | [-3.21, 0.79] | -0.07 | [-0.19, 0.05] | .00 | [-.01, .02] | -.12* |  |  |
| STMO | -0.75 | [-1.94, 0.44] | -0.07 | [-0.19, 0.04] | .00 | [-.01, .02] | -.02 |  |  |
|  |  |  |  |  |  |  |  | *R^2^*  = .043** | Δ*R^2^*  = .008 |
|  |  |  |  |  |  |  |  | 95% CI[.00,.09] | 95% CI[-.01, .03] |
|  |  |  |  |  |  |  |  |  |  |
| (Intercept) | 41.13** | [27.75, 54.50] |  |  |  |  |  |  |  |
| LH K-Factor | -4.72** | [-7.97, -1.48] | -0.17 | [-0.30, -0.05] | .03 | [-.01, .06] | -.19** |  |  |
| LTMO | -1.25 | [-3.28, 0.78] | -0.07 | [-0.20, 0.05] | .00 | [-.01, .02] | -.12* |  |  |
| STMO | -0.75 | [-2.00, 0.49] | -0.07 | [-0.19, 0.05] | .00 | [-.01, .02] | -.02 |  |  |
| Sex Partners | 0.02 | [-0.21, 0.25] | 0.02 | [-0.29, 0.34] | .00 | [-.00, .00] | -.01 |  |  |
| Sex Partners (One-Time) | -0.03 | [-0.31, 0.26] | -0.03 | [-0.34, 0.28] | .00 | [-.00, .00] | .00 |  |  |
|  |  |  |  |  |  |  |  | *R^2^*  = .043* | Δ*R^2^*  = .000 |
|  |  |  |  |  |  |  |  | 95% CI[.00,.08] | 95% CI[-.00, .00] |
|  |  |  |  |  |  |  |  |  |  |
| (Intercept) | 48.90** | [33.31, 64.49] |  |  |  |  |  |  |  |
| LH K-Factor | -4.36** | [-7.61, -1.10] | -0.16 | [-0.28, -0.04] | .02 | [-.01, .05] | -.19** |  |  |
| LTMO | -1.20 | [-3.23, 0.83] | -0.07 | [-0.19, 0.05] | .00 | [-.01, .02] | -.12* |  |  |
| STMO | -0.91 | [-2.16, 0.34] | -0.09 | [-0.21, 0.03] | .01 | [-.01, .02] | -.02 |  |  |
| Sex Partners | 0.00 | [-0.23, 0.23] | 0.00 | [-0.31, 0.31] | .00 | [-.00, .00] | -.01 |  |  |
| Sex Partners (One-Time) | -0.01 | [-0.29, 0.28] | -0.01 | [-0.32, 0.30] | .00 | [-.00, .00] | .00 |  |  |
| HVS-M | -2.19* | [-4.37, -0.01] | -0.12 | [-0.23, -0.00] | .01 | [-.01, .04] | -.12* |  |  |
| HVS-P | 0.03 | [-0.09, 0.14] | 0.03 | [-0.09, 0.14] | .00 | [-.00, .01] | .01 |  |  |
|  |  |  |  |  |  |  |  | *R^2^*  = .055* | Δ*R^2^*  = .012 |
|  |  |  |  |  |  |  |  | 95% CI[.00,.09] | 95% CI[-.01, .04] |
|  |  |  |  |  |  |  |  |  |  |
| (Intercept) | 63.94** | [44.87, 83.00] |  |  |  |  |  |  |  |
| LH K-Factor | -3.65* | [-6.98, -0.32] | -0.14 | [-0.26, -0.01] | .01 | [-.01, .04] | -.19** |  |  |
| LTMO | -1.47 | [-3.50, 0.55] | -0.09 | [-0.21, 0.03] | .01 | [-.01, .02] | -.12* |  |  |
| STMO | -0.44 | [-1.84, 0.96] | -0.04 | [-0.18, 0.09] | .00 | [-.01, .01] | -.02 |  |  |
| Sex Partners | 0.01 | [-0.23, 0.24] | 0.01 | [-0.32, 0.33] | .00 | [-.00, .00] | -.01 |  |  |
| Sex Partners (One-Time) | 0.00 | [-0.29, 0.30] | 0.00 | [-0.32, 0.32] | .00 | [-.00, .00] | .00 |  |  |
| HVS-M | -2.00 | [-4.22, 0.23] | -0.11 | [-0.22, 0.01] | .01 | [-.01, .03] | -.12* |  |  |
| HVS-P | -0.01 | [-0.12, 0.10] | -0.01 | [-0.12, 0.10] | .00 | [-.00, .00] | .01 |  |  |
| Gender | -5.83* | [-11.39, -0.28] | -0.14 | [-0.27, -0.01] | .01 | [-.01, .04] | -.10 |  |  |
| Age | -0.27* | [-0.48, -0.06] | -0.15 | [-0.27, -0.03] | .02 | [-.01, .05] | -.15** |  |  |
| Income | -0.44 | [-2.98, 2.10] | -0.02 | [-0.14, 0.10] | .00 | [-.00, .00] | -.12* |  |  |
| Education | -0.73 | [-2.30, 0.84] | -0.05 | [-0.17, 0.06] | .00 | [-.01, .01] | -.08 |  |  |
| Parenthood | -1.22 | [-6.39, 3.94] | -0.03 | [-0.15, 0.09] | .00 | [-.00, .01] | -.03 |  |  |
|  |  |  |  |  |  |  |  | *R^2^*  = .093** | Δ*R^2^*  = .038* |
|  |  |  |  |  |  |  |  | 95% CI[.01,.12] | 95% CI[-.00, .08] |
|  |  |  |  |  |  |  |  |  |  |

**Table S10. Study 2: Hierarchical regression results using Sleep Duration Variability as the criterion**

* indicates *p* < .05. ** indicates *p* < .01. Gender was coded as female = 0, male = 1. Income was z-scored to provide an interpretable unstandardized coefficient (*b*).

| Predictor | *b* | *b*  95% CI  [LL, UL] | *beta* | *beta*  95% CI  [LL, UL] | *sr^2^* | *sr^2^*  95% CI  [LL, UL] | *r* | Fit | Difference |
| --- | --- | --- | --- | --- | --- | --- | --- | --- | --- |
| (Intercept) | 80.53** | [71.51, 89.55] |  |  |  |  |  |  |  |
| LH K-Factor | -10.39** | [-17.39, -3.39] | -0.17 | [-0.28, -0.05] | .03 | [.00, .07] | -.17** |  |  |
|  |  |  |  |  |  |  |  | *R^2^*  = .027** |  |
|  |  |  |  |  |  |  |  | 95% CI[.00,.07] |  |
|  |  |  |  |  |  |  |  |  |  |
| (Intercept) | 76.97** | [46.16, 107.78] |  |  |  |  |  |  |  |
| LH K-Factor | -8.29* | [-15.81, -0.77] | -0.13 | [-0.25, -0.01] | .01 | [-.01, .04] | -.17** |  |  |
| LTMO | -1.27 | [-5.91, 3.38] | -0.03 | [-0.15, 0.09] | .00 | [-.01, .01] | -.10 |  |  |
| STMO | 2.75 | [-0.02, 5.51] | 0.11 | [-0.00, 0.23] | .01 | [-.01, .04] | .15* |  |  |
|  |  |  |  |  |  |  |  | *R^2^*  = .042** | Δ*R^2^*  = .014 |
|  |  |  |  |  |  |  |  | 95% CI[.00,.09] | 95% CI[-.01, .04] |
|  |  |  |  |  |  |  |  |  |  |
| (Intercept) | 79.47** | [48.46, 110.48] |  |  |  |  |  |  |  |
| LH K-Factor | -8.47* | [-16.00, -0.95] | -0.14 | [-0.25, -0.02] | .02 | [-.01, .04] | -.17** |  |  |
| LTMO | -1.74 | [-6.46, 2.97] | -0.05 | [-0.17, 0.08] | .00 | [-.01, .01] | -.10 |  |  |
| STMO | 3.01* | [0.13, 5.90] | 0.12 | [0.01, 0.24] | .01 | [-.01, .04] | .15* |  |  |
| Sex Partners | 0.10 | [-0.43, 0.64] | 0.06 | [-0.25, 0.37] | .00 | [-.00, .01] | -.02 |  |  |
| Sex Partners (One-Time) | -0.30 | [-0.96, 0.36] | -0.14 | [-0.45, 0.17] | .00 | [-.01, .01] | -.04 |  |  |
|  |  |  |  |  |  |  |  | *R^2^*  = .049** | Δ*R^2^*  = .007 |
|  |  |  |  |  |  |  |  | 95% CI[.00,.09] | 95% CI[-.01, .03] |
|  |  |  |  |  |  |  |  |  |  |
| (Intercept) | 85.60** | [49.38, 121.82] |  |  |  |  |  |  |  |
| LH K-Factor | -8.54* | [-16.10, -0.97] | -0.14 | [-0.26, -0.02] | .02 | [-.01, .04] | -.17** |  |  |
| LTMO | -1.86 | [-6.58, 2.85] | -0.05 | [-0.17, 0.07] | .00 | [-.01, .01] | -.10 |  |  |
| STMO | 2.93* | [0.03, 5.84] | 0.12 | [0.00, 0.24] | .01 | [-.01, .04] | .15* |  |  |
| Sex Partners | 0.11 | [-0.43, 0.64] | 0.06 | [-0.25, 0.38] | .00 | [-.00, .01] | -.02 |  |  |
| Sex Partners (One-Time) | -0.31 | [-0.97, 0.36] | -0.14 | [-0.45, 0.17] | .00 | [-.01, .01] | -.04 |  |  |
| HVS-M | -0.67 | [-5.73, 4.40] | -0.02 | [-0.13, 0.10] | .00 | [-.00, .00] | -.07 |  |  |
| HVS-P | -0.19 | [-0.46, 0.07] | -0.08 | [-0.20, 0.03] | .01 | [-.01, .02] | -.08 |  |  |
|  |  |  |  |  |  |  |  | *R^2^*  = .057* | Δ*R^2^*  = .008 |
|  |  |  |  |  |  |  |  | 95% CI[.00,.09] | 95% CI[-.01, .03] |
|  |  |  |  |  |  |  |  |  |  |
| (Intercept) | 92.60** | [47.78, 137.41] |  |  |  |  |  |  |  |
| LH K-Factor | -8.29* | [-16.12, -0.47] | -0.13 | [-0.26, -0.01] | .01 | [-.01, .04] | -.17** |  |  |
| LTMO | -2.32 | [-7.09, 2.44] | -0.06 | [-0.18, 0.06] | .00 | [-.01, .01] | -.10 |  |  |
| STMO | 3.53* | [0.23, 6.83] | 0.15 | [0.01, 0.28] | .01 | [-.01, .04] | .15* |  |  |
| Sex Partners | 0.12 | [-0.44, 0.68] | 0.07 | [-0.26, 0.40] | .00 | [-.00, .01] | -.02 |  |  |
| Sex Partners (One-Time) | -0.32 | [-1.01, 0.37] | -0.15 | [-0.47, 0.17] | .00 | [-.01, .01] | -.04 |  |  |
| HVS-M | -0.47 | [-5.71, 4.76] | -0.01 | [-0.13, 0.11] | .00 | [-.00, .00] | -.07 |  |  |
| HVS-P | -0.23 | [-0.50, 0.04] | -0.10 | [-0.22, 0.02] | .01 | [-.01, .03] | -.08 |  |  |
| Gender | -4.07 | [-17.13, 8.99] | -0.04 | [-0.17, 0.09] | .00 | [-.01, .01] | .03 |  |  |
| Age | -0.15 | [-0.65, 0.34] | -0.04 | [-0.16, 0.08] | .00 | [-.01, .01] | -.07 |  |  |
| Income | -3.02 | [-8.99, 2.95] | -0.06 | [-0.18, 0.06] | .00 | [-.01, .02] | -.09 |  |  |
| Education | -0.65 | [-4.34, 3.04] | -0.02 | [-0.14, 0.10] | .00 | [-.00, .00] | -.04 |  |  |
| Parenthood | 8.75 | [-3.39, 20.90] | 0.09 | [-0.03, 0.21] | .01 | [-.01, .02] | .04 |  |  |
|  |  |  |  |  |  |  |  | *R^2^*  = .073* | Δ*R^2^*  = .016 |
|  |  |  |  |  |  |  |  | 95% CI[.00,.10] | 95% CI[-.01, .04] |
|  |  |  |  |  |  |  |  |  |  |

**Table S11. Study 2: Hierarchical regression results using Wakefulness After Sleep Onset as the criterion**

* indicates *p* < .05. ** indicates *p* < .01. Gender was coded as female = 0, male = 1. Income was z-scored to provide an interpretable unstandardized coefficient (*b*).

| Predictor | *b* | *b*  95% CI  [LL, UL] | *beta* | *beta*  95% CI  [LL, UL] | *sr^2^* | *sr^2^*  95% CI  [LL, UL] | *r* | Fit | Difference |
| --- | --- | --- | --- | --- | --- | --- | --- | --- | --- |
| (Intercept) | 27.98** | [22.43, 33.53] |  |  |  |  |  |  |  |
| LH K-Factor | -2.11 | [-6.41, 2.19] | -0.06 | [-0.17, 0.06] | .00 | [.00, .03] | -.06 |  |  |
|  |  |  |  |  |  |  |  | *R^2^*  = .003 |  |
|  |  |  |  |  |  |  |  | 95% CI[.00,.03] |  |
|  |  |  |  |  |  |  |  |  |  |
| (Intercept) | 48.98** | [30.05, 67.90] |  |  |  |  |  |  |  |
| LH K-Factor | -0.93 | [-5.55, 3.69] | -0.02 | [-0.15, 0.10] | .00 | [-.00, .01] | -.06 |  |  |
| LTMO | -3.00* | [-5.86, -0.15] | -0.13 | [-0.25, -0.01] | .01 | [-.01, .04] | -.12* |  |  |
| STMO | -1.09 | [-2.79, 0.61] | -0.07 | [-0.19, 0.04] | .01 | [-.01, .02] | -.04 |  |  |
|  |  |  |  |  |  |  |  | *R^2^*  = .020 | Δ*R^2^*  = .017 |
|  |  |  |  |  |  |  |  | 95% CI[.00,.05] | 95% CI[-.01, .05] |
|  |  |  |  |  |  |  |  |  |  |
| (Intercept) | 49.13** | [30.07, 68.19] |  |  |  |  |  |  |  |
| LH K-Factor | -1.00 | [-5.63, 3.62] | -0.03 | [-0.15, 0.09] | .00 | [-.00, .01] | -.06 |  |  |
| LTMO | -3.09* | [-5.98, -0.19] | -0.13 | [-0.25, -0.01] | .01 | [-.01, .04] | -.12* |  |  |
| STMO | -1.44 | [-3.22, 0.33] | -0.10 | [-0.22, 0.02] | .01 | [-.01, .03] | -.04 |  |  |
| Sex Partners | 0.19 | [-0.14, 0.51] | 0.18 | [-0.14, 0.49] | .00 | [-.01, .02] | .06 |  |  |
| Sex Partners (One-Time) | -0.15 | [-0.56, 0.26] | -0.11 | [-0.43, 0.20] | .00 | [-.01, .01] | .05 |  |  |
|  |  |  |  |  |  |  |  | *R^2^*  = .026 | Δ*R^2^*  = .006 |
|  |  |  |  |  |  |  |  | 95% CI[.00,.06] | 95% CI[-.01, .02] |
|  |  |  |  |  |  |  |  |  |  |
| (Intercept) | 46.00** | [23.70, 68.31] |  |  |  |  |  |  |  |
| LH K-Factor | -1.26 | [-5.92, 3.41] | -0.03 | [-0.16, 0.09] | .00 | [-.01, .01] | -.06 |  |  |
| LTMO | -3.15* | [-6.06, -0.25] | -0.13 | [-0.26, -0.01] | .01 | [-.01, .04] | -.12* |  |  |
| STMO | -1.37 | [-3.16, 0.42] | -0.09 | [-0.21, 0.03] | .01 | [-.01, .03] | -.04 |  |  |
| Sex Partners | 0.20 | [-0.13, 0.53] | 0.19 | [-0.13, 0.51] | .00 | [-.01, .02] | .06 |  |  |
| Sex Partners (One-Time) | -0.16 | [-0.57, 0.24] | -0.13 | [-0.44, 0.19] | .00 | [-.01, .01] | .05 |  |  |
| HVS-M | 1.20 | [-1.92, 4.32] | 0.05 | [-0.07, 0.16] | .00 | [-.01, .01] | .03 |  |  |
| HVS-P | -0.07 | [-0.24, 0.09] | -0.05 | [-0.17, 0.06] | .00 | [-.01, .01] | -.04 |  |  |
|  |  |  |  |  |  |  |  | *R^2^*  = .030 | Δ*R^2^*  = .004 |
|  |  |  |  |  |  |  |  | 95% CI[.00,.05] | 95% CI[-.01, .02] |
|  |  |  |  |  |  |  |  |  |  |
| (Intercept) | 61.48** | [34.78, 88.18] |  |  |  |  |  |  |  |
| LH K-Factor | -1.23 | [-5.89, 3.44] | -0.03 | [-0.15, 0.09] | .00 | [-.01, .01] | -.06 |  |  |
| LTMO | -3.33* | [-6.17, -0.49] | -0.14 | [-0.26, -0.02] | .02 | [-.01, .04] | -.12* |  |  |
| STMO | 0.56 | [-1.41, 2.52] | 0.04 | [-0.10, 0.17] | .00 | [-.01, .01] | -.04 |  |  |
| Sex Partners | 0.04 | [-0.29, 0.38] | 0.04 | [-0.28, 0.36] | .00 | [-.00, .00] | .06 |  |  |
| Sex Partners (One-Time) | 0.02 | [-0.39, 0.43] | 0.02 | [-0.30, 0.33] | .00 | [-.00, .00] | .05 |  |  |
| HVS-M | 0.03 | [-3.09, 3.15] | 0.00 | [-0.12, 0.12] | .00 | [-.00, .00] | .03 |  |  |
| HVS-P | -0.12 | [-0.28, 0.04] | -0.09 | [-0.20, 0.03] | .01 | [-.01, .02] | -.04 |  |  |
| Gender | -15.75** | [-23.53, -7.97] | -0.26 | [-0.39, -0.13] | .05 | [.00, .09] | -.23** |  |  |
| Age | -0.02 | [-0.31, 0.28] | -0.01 | [-0.13, 0.11] | .00 | [-.00, .00] | .03 |  |  |
| Income | 1.12 | [-2.44, 4.68] | 0.04 | [-0.08, 0.15] | .00 | [-.01, .01] | -.03 |  |  |
| Education | -2.56* | [-4.75, -0.36] | -0.13 | [-0.25, -0.02] | .02 | [-.01, .04] | -.13* |  |  |
| Parenthood | 4.66 | [-2.58, 11.89] | 0.08 | [-0.04, 0.19] | .00 | [-.01, .02] | .08 |  |  |
|  |  |  |  |  |  |  |  | *R^2^*  = .108** | Δ*R^2^*  = .078** |
|  |  |  |  |  |  |  |  | 95% CI[.02,.14] | 95% CI[.02, .13] |
|  |  |  |  |  |  |  |  |  |  |

**Table S12. Study 2: Hierarchical regression results using Eveningness as the criterion**

* indicates *p* < .05. ** indicates *p* < .01. Gender was coded as female = 0, male = 1. Income was z-scored to provide an interpretable unstandardized coefficient (*b*).

| Predictor | *b* | *b*  95% CI  [LL, UL] | *beta* | *beta*  95% CI  [LL, UL] | *sr^2^* | *sr^2^*  95% CI  [LL, UL] | *r* | Fit | Difference |
| --- | --- | --- | --- | --- | --- | --- | --- | --- | --- |
| (Intercept) | 7.16** | [6.67, 7.65] |  |  |  |  |  |  |  |
| LH K-Factor | -0.39* | [-0.76, -0.01] | -0.11 | [-0.23, -0.00] | .01 | [.00, .05] | -.11* |  |  |
|  |  |  |  |  |  |  |  | *R^2^*  = .013* |  |
|  |  |  |  |  |  |  |  | 95% CI[.00,.05] |  |
|  |  |  |  |  |  |  |  |  |  |
| (Intercept) | 5.41** | [3.74, 7.07] |  |  |  |  |  |  |  |
| LH K-Factor | -0.47* | [-0.87, -0.06] | -0.14 | [-0.26, -0.02] | .02 | [-.01, .04] | -.11* |  |  |
| LTMO | 0.24 | [-0.01, 0.49] | 0.11 | [-0.01, 0.24] | .01 | [-.01, .03] | .05 |  |  |
| STMO | 0.11 | [-0.04, 0.26] | 0.08 | [-0.03, 0.20] | .01 | [-.01, .02] | .09 |  |  |
|  |  |  |  |  |  |  |  | *R^2^*  = .029* | Δ*R^2^*  = .016 |
|  |  |  |  |  |  |  |  | 95% CI[.00,.07] | 95% CI[-.01, .04] |
|  |  |  |  |  |  |  |  |  |  |
| (Intercept) | 5.72** | [4.09, 7.35] |  |  |  |  |  |  |  |
| LH K-Factor | -0.49* | [-0.88, -0.09] | -0.14 | [-0.26, -0.03] | .02 | [-.01, .05] | -.11* |  |  |
| LTMO | 0.18 | [-0.07, 0.43] | 0.09 | [-0.03, 0.21] | .01 | [-.01, .02] | .05 |  |  |
| STMO | 0.17* | [0.02, 0.32] | 0.13 | [0.01, 0.25] | .01 | [-.01, .04] | .09 |  |  |
| Sex Partners | 0.00 | [-0.03, 0.03] | 0.01 | [-0.30, 0.32] | .00 | [-.00, .00] | -.19** |  |  |
| Sex Partners (One-Time) | -0.03 | [-0.06, 0.01] | -0.25 | [-0.56, 0.05] | .01 | [-.01, .03] | -.22** |  |  |
|  |  |  |  |  |  |  |  | *R^2^*  = .085** | Δ*R^2^*  = .056** |
|  |  |  |  |  |  |  |  | 95% CI[.02,.14] | 95% CI[.01, .11] |
|  |  |  |  |  |  |  |  |  |  |
| (Intercept) | 7.14** | [5.27, 9.01] |  |  |  |  |  |  |  |
| LH K-Factor | -0.40* | [-0.79, -0.01] | -0.12 | [-0.24, -0.00] | .01 | [-.01, .03] | -.11* |  |  |
| LTMO | 0.20 | [-0.04, 0.44] | 0.10 | [-0.02, 0.21] | .01 | [-.01, .03] | .05 |  |  |
| STMO | 0.14 | [-0.01, 0.29] | 0.11 | [-0.01, 0.22] | .01 | [-.01, .03] | .09 |  |  |
| Sex Partners | -0.00 | [-0.03, 0.02] | -0.03 | [-0.33, 0.27] | .00 | [-.00, .00] | -.19** |  |  |
| Sex Partners (One-Time) | -0.02 | [-0.06, 0.01] | -0.21 | [-0.51, 0.09] | .01 | [-.01, .02] | -.22** |  |  |
| HVS-M | -0.45** | [-0.72, -0.19] | -0.19 | [-0.31, -0.08] | .03 | [-.00, .07] | -.19** |  |  |
| HVS-P | 0.02* | [0.00, 0.03] | 0.13 | [0.02, 0.24] | .02 | [-.01, .04] | .09 |  |  |
|  |  |  |  |  |  |  |  | *R^2^*  = .127** | Δ*R^2^*  = .042** |
|  |  |  |  |  |  |  |  | 95% CI[.05,.18] | 95% CI[-.00, .08] |
|  |  |  |  |  |  |  |  |  |  |
| (Intercept) | 9.89** | [7.67, 12.11] |  |  |  |  |  |  |  |
| LH K-Factor | -0.24 | [-0.63, 0.15] | -0.07 | [-0.19, 0.04] | .00 | [-.01, .02] | -.11* |  |  |
| LTMO | 0.16 | [-0.08, 0.39] | 0.08 | [-0.04, 0.19] | .00 | [-.01, .02] | .05 |  |  |
| STMO | 0.04 | [-0.12, 0.20] | 0.03 | [-0.09, 0.16] | .00 | [-.00, .01] | .09 |  |  |
| Sex Partners | 0.01 | [-0.01, 0.04] | 0.14 | [-0.16, 0.45] | .00 | [-.01, .01] | -.19** |  |  |
| Sex Partners (One-Time) | -0.04* | [-0.07, -0.01] | -0.34 | [-0.64, -0.05] | .01 | [-.01, .04] | -.22** |  |  |
| HVS-M | -0.34** | [-0.60, -0.09] | -0.15 | [-0.26, -0.04] | .02 | [-.01, .05] | -.19** |  |  |
| HVS-P | 0.01* | [0.00, 0.03] | 0.11 | [0.00, 0.22] | .01 | [-.01, .03] | .09 |  |  |
| Gender | 0.43 | [-0.22, 1.07] | 0.08 | [-0.04, 0.20] | .00 | [-.01, .02] | .12* |  |  |
| Age | -0.05** | [-0.08, -0.03] | -0.24 | [-0.36, -0.13] | .05 | [.01, .09] | -.29** |  |  |
| Income | -0.09 | [-0.39, 0.20] | -0.04 | [-0.15, 0.08] | .00 | [-.01, .01] | -.12* |  |  |
| Education | -0.20* | [-0.38, -0.02] | -0.12 | [-0.22, -0.01] | .01 | [-.01, .03] | -.13* |  |  |
| Parenthood | -0.60* | [-1.20, -0.00] | -0.11 | [-0.22, -0.00] | .01 | [-.01, .03] | -.10 |  |  |
|  |  |  |  |  |  |  |  | *R^2^*  = .212** | Δ*R^2^*  = .085** |
|  |  |  |  |  |  |  |  | 95% CI[.11,.26] | 95% CI[.03, .14] |
|  |  |  |  |  |  |  |  |  |  |

**Table S13. Study 2: Hierarchical regression results using Sleep-Wake Stability as the criterion**

* indicates *p* < .05. ** indicates *p* < .01. Gender was coded as female = 0, male = 1. Income was z-scored to provide an interpretable unstandardized coefficient (*b*).

| Predictor | *b* | *b*  95% CI  [LL, UL] | *beta* | *beta*  95% CI  [LL, UL] | *sr^2^* | *sr^2^*  95% CI  [LL, UL] | *r* | Fit | Difference |
| --- | --- | --- | --- | --- | --- | --- | --- | --- | --- |
| (Intercept) | 7.98** | [7.64, 8.32] |  |  |  |  |  |  |  |
| LH K-Factor | 0.31* | [0.05, 0.57] | 0.13 | [0.02, 0.25] | .02 | [.00, .06] | .13* |  |  |
|  |  |  |  |  |  |  |  | *R^2^*  = .018* |  |
|  |  |  |  |  |  |  |  | 95% CI[.00,.06] |  |
|  |  |  |  |  |  |  |  |  |  |
| (Intercept) | 8.05** | [6.90, 9.20] |  |  |  |  |  |  |  |
| LH K-Factor | 0.22 | [-0.06, 0.50] | 0.10 | [-0.02, 0.22] | .01 | [-.01, .03] | .13* |  |  |
| LTMO | 0.06 | [-0.11, 0.23] | 0.04 | [-0.08, 0.16] | .00 | [-.01, .01] | .10 |  |  |
| STMO | -0.11* | [-0.21, -0.00] | -0.12 | [-0.23, -0.00] | .01 | [-.01, .04] | -.14* |  |  |
|  |  |  |  |  |  |  |  | *R^2^*  = .034* | Δ*R^2^*  = .016 |
|  |  |  |  |  |  |  |  | 95% CI[.00,.08] | 95% CI[-.01, .04] |
|  |  |  |  |  |  |  |  |  |  |
| (Intercept) | 7.92** | [6.77, 9.07] |  |  |  |  |  |  |  |
| LH K-Factor | 0.23 | [-0.05, 0.51] | 0.10 | [-0.02, 0.22] | .01 | [-.01, .03] | .13* |  |  |
| LTMO | 0.09 | [-0.09, 0.26] | 0.06 | [-0.06, 0.18] | .00 | [-.01, .02] | .10 |  |  |
| STMO | -0.12* | [-0.22, -0.01] | -0.13 | [-0.25, -0.01] | .01 | [-.01, .04] | -.14* |  |  |
| Sex Partners | -0.01 | [-0.03, 0.01] | -0.12 | [-0.43, 0.20] | .00 | [-.01, .01] | .04 |  |  |
| Sex Partners (One-Time) | 0.02 | [-0.01, 0.04] | 0.22 | [-0.09, 0.53] | .01 | [-.01, .02] | .06 |  |  |
|  |  |  |  |  |  |  |  | *R^2^*  = .047* | Δ*R^2^*  = .013 |
|  |  |  |  |  |  |  |  | 95% CI[.00,.09] | 95% CI[-.01, .04] |
|  |  |  |  |  |  |  |  |  |  |
| (Intercept) | 6.51** | [5.21, 7.82] |  |  |  |  |  |  |  |
| LH K-Factor | 0.17 | [-0.11, 0.44] | 0.07 | [-0.05, 0.19] | .00 | [-.01, .02] | .13* |  |  |
| LTMO | 0.08 | [-0.09, 0.25] | 0.05 | [-0.06, 0.17] | .00 | [-.01, .01] | .10 |  |  |
| STMO | -0.09 | [-0.19, 0.02] | -0.10 | [-0.21, 0.02] | .01 | [-.01, .03] | -.14* |  |  |
| Sex Partners | -0.00 | [-0.02, 0.01] | -0.07 | [-0.37, 0.23] | .00 | [-.00, .01] | .04 |  |  |
| Sex Partners (One-Time) | 0.01 | [-0.01, 0.04] | 0.17 | [-0.14, 0.47] | .00 | [-.01, .02] | .06 |  |  |
| HVS-M | 0.40** | [0.22, 0.58] | 0.25 | [0.13, 0.36] | .06 | [.01, .10] | .26** |  |  |
| HVS-P | -0.01 | [-0.01, 0.00] | -0.06 | [-0.17, 0.05] | .00 | [-.01, .02] | -.02 |  |  |
|  |  |  |  |  |  |  |  | *R^2^*  = .103** | Δ*R^2^*  = .056** |
|  |  |  |  |  |  |  |  | 95% CI[.03,.15] | 95% CI[.01, .10] |
|  |  |  |  |  |  |  |  |  |  |
| (Intercept) | 5.22** | [3.61, 6.82] |  |  |  |  |  |  |  |
| LH K-Factor | 0.08 | [-0.19, 0.36] | 0.04 | [-0.08, 0.16] | .00 | [-.01, .01] | .13* |  |  |
| LTMO | 0.09 | [-0.08, 0.27] | 0.07 | [-0.05, 0.18] | .00 | [-.01, .02] | .10 |  |  |
| STMO | -0.08 | [-0.20, 0.04] | -0.09 | [-0.22, 0.04] | .01 | [-.01, .02] | -.14* |  |  |
| Sex Partners | -0.01 | [-0.03, 0.01] | -0.13 | [-0.45, 0.18] | .00 | [-.01, .01] | .04 |  |  |
| Sex Partners (One-Time) | 0.02 | [-0.01, 0.04] | 0.21 | [-0.10, 0.52] | .01 | [-.01, .02] | .06 |  |  |
| HVS-M | 0.36** | [0.17, 0.55] | 0.22 | [0.11, 0.34] | .04 | [.00, .08] | .26** |  |  |
| HVS-P | -0.00 | [-0.01, 0.01] | -0.04 | [-0.15, 0.07] | .00 | [-.01, .01] | -.02 |  |  |
| Gender | 0.16 | [-0.31, 0.63] | 0.04 | [-0.08, 0.17] | .00 | [-.01, .01] | -.06 |  |  |
| Age | 0.03** | [0.01, 0.04] | 0.17 | [0.05, 0.29] | .02 | [-.01, .06] | .20** |  |  |
| Income | 0.03 | [-0.18, 0.25] | 0.02 | [-0.10, 0.13] | .00 | [-.00, .00] | .10 |  |  |
| Education | 0.06 | [-0.07, 0.19] | 0.05 | [-0.06, 0.16] | .00 | [-.01, .01] | .05 |  |  |
| Parenthood | 0.36 | [-0.07, 0.80] | 0.10 | [-0.02, 0.21] | .01 | [-.01, .03] | .11 |  |  |
|  |  |  |  |  |  |  |  | *R^2^*  = .137** | Δ*R^2^*  = .034* |
|  |  |  |  |  |  |  |  | 95% CI[.04,.18] | 95% CI[-.00, .07] |
|  |  |  |  |  |  |  |  |  |  |

**Table S14. Study 2: Hierarchical regression results using Hypothetical Sleep Duration Variability as the criterion**

* indicates *p* < .05. ** indicates *p* < .01. Gender was coded as female = 0, male = 1. Income was z-scored to provide an interpretable unstandardized coefficient (*b*).

| Predictor | *b* | *b*  95% CI  [LL, UL] | *beta* | *beta*  95% CI  [LL, UL] | *sr^2^* | *sr^2^*  95% CI  [LL, UL] | *r* | Fit | Difference |
| --- | --- | --- | --- | --- | --- | --- | --- | --- | --- |
| (Intercept) | 255.77** | [233.94, 277.60] |  |  |  |  |  |  |  |
| LH K-Factor | -23.68** | [-40.61, -6.74] | -0.16 | [-0.27, -0.04] | .02 | [.00, .07] | -.16** |  |  |
|  |  |  |  |  |  |  |  | *R^2^*  = .024** |  |
|  |  |  |  |  |  |  |  | 95% CI[.00,.07] |  |
|  |  |  |  |  |  |  |  |  |  |
| (Intercept) | 175.58** | [102.35, 248.82] |  |  |  |  |  |  |  |
| LH K-Factor | -20.07* | [-37.95, -2.20] | -0.13 | [-0.25, -0.01] | .02 | [-.01, .04] | -.16** |  |  |
| LTMO | 5.20 | [-5.85, 16.26] | 0.06 | [-0.06, 0.17] | .00 | [-.01, .01] | -.04 |  |  |
| STMO | 13.20** | [6.62, 19.78] | 0.23 | [0.11, 0.34] | .05 | [.00, .09] | .24** |  |  |
|  |  |  |  |  |  |  |  | *R^2^*  = .073** | Δ*R^2^*  = .048** |
|  |  |  |  |  |  |  |  | 95% CI[.02,.13] | 95% CI[.00, .09] |
|  |  |  |  |  |  |  |  |  |  |
| (Intercept) | 182.46** | [109.10, 255.81] |  |  |  |  |  |  |  |
| LH K-Factor | -20.43* | [-38.23, -2.64] | -0.13 | [-0.25, -0.02] | .02 | [-.01, .04] | -.16** |  |  |
| LTMO | 4.01 | [-7.14, 15.15] | 0.04 | [-0.08, 0.16] | .00 | [-.01, .01] | -.04 |  |  |
| STMO | 14.78** | [7.96, 21.60] | 0.25 | [0.14, 0.37] | .06 | [.01, .10] | .24** |  |  |
| Sex Partners | -0.12 | [-1.38, 1.14] | -0.03 | [-0.33, 0.28] | .00 | [-.00, .00] | -.05 |  |  |
| Sex Partners (One-Time) | -0.54 | [-2.10, 1.03] | -0.10 | [-0.41, 0.20] | .00 | [-.01, .01] | -.07 |  |  |
|  |  |  |  |  |  |  |  | *R^2^*  = .089** | Δ*R^2^*  = .016 |
|  |  |  |  |  |  |  |  | 95% CI[.03,.14] | 95% CI[-.01, .04] |
|  |  |  |  |  |  |  |  |  |  |
| (Intercept) | 235.43** | [150.24, 320.62] |  |  |  |  |  |  |  |
| LH K-Factor | -18.08* | [-35.88, -0.27] | -0.12 | [-0.24, -0.00] | .01 | [-.01, .04] | -.16** |  |  |
| LTMO | 4.28 | [-6.81, 15.36] | 0.05 | [-0.07, 0.16] | .00 | [-.01, .01] | -.04 |  |  |
| STMO | 13.73** | [6.90, 20.56] | 0.23 | [0.12, 0.35] | .05 | [.00, .09] | .24** |  |  |
| Sex Partners | -0.22 | [-1.48, 1.04] | -0.05 | [-0.36, 0.25] | .00 | [-.00, .00] | -.05 |  |  |
| Sex Partners (One-Time) | -0.40 | [-1.96, 1.16] | -0.08 | [-0.38, 0.23] | .00 | [-.01, .01] | -.07 |  |  |
| HVS-M | -14.51* | [-26.42, -2.59] | -0.14 | [-0.25, -0.02] | .02 | [-.01, .04] | -.18** |  |  |
| HVS-P | 0.10 | [-0.52, 0.72] | 0.02 | [-0.09, 0.13] | .00 | [-.00, .00] | -.01 |  |  |
|  |  |  |  |  |  |  |  | *R^2^*  = .106** | Δ*R^2^*  = .017 |
|  |  |  |  |  |  |  |  | 95% CI[.03,.16] | 95% CI[-.01, .05] |
|  |  |  |  |  |  |  |  |  |  |
| (Intercept) | 307.11** | [203.28, 410.94] |  |  |  |  |  |  |  |
| LH K-Factor | -12.38 | [-30.51, 5.75] | -0.08 | [-0.20, 0.04] | .01 | [-.01, .02] | -.16** |  |  |
| LTMO | 2.61 | [-8.44, 13.65] | 0.03 | [-0.09, 0.15] | .00 | [-.00, .01] | -.04 |  |  |
| STMO | 12.53** | [4.89, 20.17] | 0.21 | [0.08, 0.34] | .03 | [-.01, .07] | .24** |  |  |
| Sex Partners | 0.21 | [-1.09, 1.51] | 0.05 | [-0.26, 0.36] | .00 | [-.00, .00] | -.05 |  |  |
| Sex Partners (One-Time) | -0.82 | [-2.42, 0.77] | -0.16 | [-0.47, 0.15] | .00 | [-.01, .01] | -.07 |  |  |
| HVS-M | -10.51 | [-22.64, 1.62] | -0.10 | [-0.21, 0.02] | .01 | [-.01, .03] | -.18** |  |  |
| HVS-P | -0.05 | [-0.67, 0.58] | -0.01 | [-0.12, 0.10] | .00 | [-.00, .00] | -.01 |  |  |
| Gender | 1.04 | [-29.22, 31.30] | 0.00 | [-0.12, 0.13] | .00 | [-.00, .00] | .13* |  |  |
| Age | -1.72** | [-2.87, -0.57] | -0.17 | [-0.29, -0.06] | .03 | [-.01, .06] | -.23** |  |  |
| Income | -11.25 | [-25.09, 2.58] | -0.09 | [-0.21, 0.02] | .01 | [-.01, .03] | -.14* |  |  |
| Education | -2.94 | [-11.49, 5.61] | -0.04 | [-0.15, 0.07] | .00 | [-.01, .01] | -.06 |  |  |
| Parenthood | -7.52 | [-35.66, 20.61] | -0.03 | [-0.14, 0.08] | .00 | [-.01, .01] | -.05 |  |  |
|  |  |  |  |  |  |  |  | *R^2^*  = .148** | Δ*R^2^*  = .042* |
|  |  |  |  |  |  |  |  | 95% CI[.05,.19] | 95% CI[.00, .08] |
|  |  |  |  |  |  |  |  |  |  |

**Figure S6. Study 1: Exploratory Visualizations of Life History Strategy Versus Hedonic Valuations of Sleep**

Values which are *lower* on the y-axis represent *slower* LH strategies (higher K-Factor). Values on the x-axis represent z-scores. From left to right: hedonic motivation for sleeping, hedonic pleasure from sleeping, and hedonic valuations of sleep (an evenly weighted parcel constructed from the hedonic motivation and pleasure items). The fit was estimated using LOESS (Locally Estimated Scatterplot Smoothing). Shaded regions represent the 95% confidence interval.


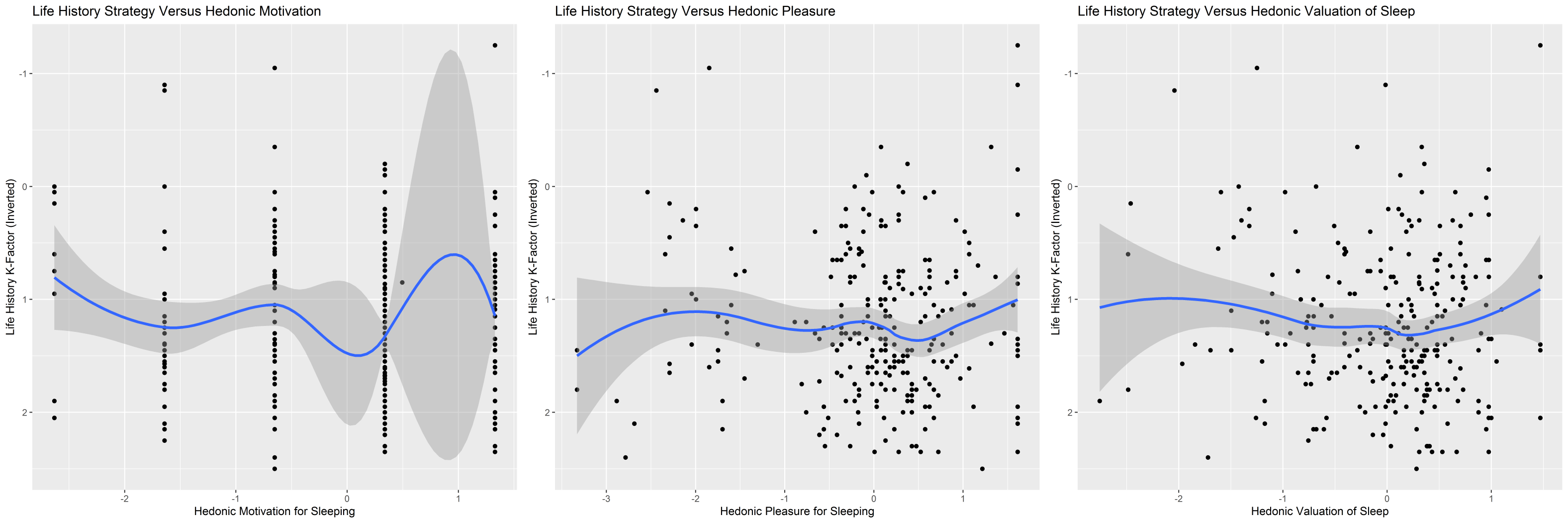

Supplement: eoaa048_Supplementary_Data [file eoaa048_supplementary_data.zip › EMPH-2020-089R1 Dishakjian et al Life History & Sleep Supplementary-Figures-and-Tables.docx]
