# Supplementary material for: Live fast, die young and sleep later: Life history strategy and human sleep behavior
Source: Evol Med Public Health. 2020 Dec 2;9(1):36–52. doi: 10.1093/emph/eoaa048 (PMC7953418; doi:10.1093/emph/eoaa048)
Supplement: eoaa048_Supplementary_Data [file eoaa048_supplementary_data.zip › EMPH-2020-089R1 Dishakjian et al Life History & Sleep-Supplementary-Methods-and-Results.docx]

**Supplementary Methods and Results for:**

**Live Fast, Die Young, and Sleep Later: Life History Strategy and Human Sleep Behavior**

Vahe Dishakjian^1,2^, Daniel M.T. Fessler^1,2,3^, and Adam Maxwell Sparks^1,2^

1. Department of Anthropology, University of California, Los Angeles
2. Center for Behavior, Evolution and Culture, University of California, Los Angeles
3. Bedari Kindness Institute, University of California, Los Angeles

**Table of Contents**

[**Methods** 2](#_Toc48245768)

[**Derivations for sleep variables using the STQ** 2](#_Toc48245769)

[***Sleep duration*** 2](#_Toc48245770)

[***Sleep-wake stability*** 2](#_Toc48245771)

[***Sleep duration variability*** 2](#_Toc48245772)

[***Hypothetical sleep duration variability*** 3](#_Toc48245773)

[***Eveningness*** 3](#_Toc48245774)

[**Statistical analyses** 4](#_Toc48245775)

[***Manual corrections for “a.m”/”p.m”*** 4](#_Toc48245776)

[***Hierarchical regression models*** 4](#_Toc48245777)

[***SEM construction*** 5](#_Toc48245778)

[**Modifications made to instruments in Study 2** 6](#_Toc48245779)

[***Sleep-wake stability*** 6](#_Toc48245780)

[***Sleep need*** 7](#_Toc48245781)

[**Results** 7](#_Toc48245782)

[***Non-linear relationships between LHS and sleep duration in Study 1*** 7](#_Toc48245783)

[***Non-linear relationships between LHS and sleep duration in Study 2*** 8](#_Toc48245784)

[***Non-linear relationships between LHS and hedonic valuations in Study 1*** 8](#_Toc48245785)

[***Non-linear relationships between LHS and hedonic valuations in Study 2*** 9](#_Toc48245786)

# **Methods**

## **Derivations for sleep variables using the STQ**

### ***Sleep duration***

Time in bed (TIB) was calculated as the difference between wake and sleep times. In total, six TIB durations were calculated: early-shifted, usual, and late-shifted TIB on a work/school day; early-shifted, usual, and late-shifted TIB on a day off (e.g. a weekend). To account for sampling fluctuations, the early-shifted, usual, and late-shifted times in bed were averaged to provide composite measures of both weekday and off day TIB. Weekday and off day TIB were weighted 5/7 and 2/7 respectively to provide a habitual measure of TIB. Sleep onset latency and wakefulness after sleep onset were then subtracted from habitual TIB to yield habitual sleep duration.

### ***Sleep-wake stability***

Weekday and weekend wake stability were weighted 5/7 and 2/7 respectively to yield a habitual measure of wake stability. Analogous procedures were undertaken to derive a habitual measure of sleep stability. Wake and sleep stability were then arithmetically averaged to provide a habitual measure of sleep-wake stability.

### ***Sleep duration variability***

This value was derived by taking the difference between the shortest and longest sleep duration of the three provided (early-shifted, usual, and late-shifted sleep duration), then assigning a 5/7 and 2/7 weighting for weekdays and off days respectively.

### ***Hypothetical sleep duration variability***

To evaluate variance in sleep behavior at the extreme tails, we calculated the disparity between participants’ hypothetical longest and shortest nights of sleep. Hypothetical longest sleep duration was quantified as the period between participants’ earliest possible bedtime and latest possible wake time. Conversely, hypothetical shortest sleep duration was quantified as the period between participants’ latest possible bedtime and earliest possible wake time. Then, we calculated the difference between the hypothetical shortest and longest nights of sleep. The weekday difference was weighted 5/7 and the off day difference was weighted 2/7 to create a composite measure of hypothetical sleep duration variability (H-SDV).

### ***Eveningness***

The STQ presents participants with the following instructions, “Please think of GOOD NIGHT TIME as the time at which you are finally in bed and trying to fall asleep.” In total, six bedtimes are recorded: earliest, usual, and latest bedtimes on a work/school day; earliest, usual, and latest sleep times on a day off (e.g. a weekend). After prompting participants with, “Please think of GOOD MORNING TIME as the time at which you finally get out of bed and start your day,” six wake times are recorded in an analogous manner. To account for sampling fluctuations, the earliest, usual, and latest times were averaged to provide composite measures of bedtime and waketime. After being converted to continuous variables, weekday and off day times were weighted 5/7 and 2/7 respectively to provide habitual measures of bedtime and waketime. Finally, these variables were added to yield a habitual measure of eveningness.

## **Statistical analyses**

The Kirby *k* discounting rate was calculated using an automated scorer [1]. Missing data were imputed using the missForest machine learning algorithm, a nonparametric method of multiple imputation which can handle mixed data types and non-linear interactions [2]. The estimated out-of-bag imputation error (NRMSE) was .005% in Study 1 and 3.14 x 10^-5^% in Study 2. VIF’s of regression indicators hovered at roughly ~1.50 and did not exceed 2.00 in Study 1; in Study 2, the VIF’s of lifetime and one-time sexual partner count were ~8.5, lower than our cutoff of 10 [3].

### ***Manual corrections for “a.m”/”p.m”***

To account for accidental errors in the manual inputting of “a.m.” and “p.m.,” we constructed tables of data points indicating negative habitual sleep durations, or durations exceeding 12 hours. Since we asked participants to provide six bedtimes and six wake times, it was possible to cross-reference the suspected typos with other wake/bedtimes to check for congruency. We corrected the “a.m.” and “p.m.” designations of only those entries which were deemed to be erroneous beyond reasonable doubt. A full list of suspected typos and corrections can be found on the OSF page for this study (<https://osf.io/kgvyt/>).

### ***Hierarchical regression models***

For the first three steps of the nested regression models, indicators were added in the order of their appositional placement on the developmental timeline of LH expression: overarching LH strategy (childhood), followed by mating orientation (adolescence), followed by mating outcomes (adulthood). Like a series of shrinking concentric circles, each successive step narrows the range of analysis to a more specific subset of coordinated LH behavior. The first step regressed sleep parameters on LH strategy only; this indicator is afforded primacy because it precedes sociosexual orientation/behavior on the developmental timeline, and is thus expected to superintend the widest range of LH-attendant behavior. In the second step, short-term and long-term mating orientation were added as indicators, since they proximally *succeed* LH strategy and *precede* sexual behavior on the timeline of LH expression. In the third step, sexual partners (lifetime and one-time) were added, since these indicators are—relative to both LH strategy and sociosexual orientation—terminal expressions of LH behavior. The fourth and final regression step includes the addition of hedonic motivation/pleasure indicators; the goal of this step was to investigate if hedonic attributions of sleep could potentially mediate or moderate the aggregate effect of LH process and outcome variables on the given sleep parameter of interest.

### ***SEM construction***

LH strategy was arranged as a purely exogenous latent variable with all paths leading *to*—rather than *from*—endogenous latent variables in the domains of maintenance and reproduction. A two-factor model with LH strategy consisting of LTMO and LH K-Factor had a better fit than a three-factor model including STMO as an indicator. Given the consistent associations and theoretical rationale (hedonic reward reinforcing sleep behavior on a consistent schedule), we hypothesized that it might act as a mediator between SWS and LH strategy. The proclivity to stay up later was also hypothesized to be affected by a greater motivation to sleep, which was supported by associations observed in both studies; thus HVS-M was hypothesized to mediate LH strategy’s effects on Eveningness. Variability was allowed to covary with Stability because of the theoretical “chicken-and-egg” quandary, i.e. lack of resolving power to determine which of the two was upstream of the other. Eveningness was assumed to have downstream effects on both Variability and Stability in consideration with previous research [4], as supported by observed relationships in both studies. Detriments followed by sleepiness were arranged most terminally on the chain of LH expression, since they are the final “symptoms” of preceding variables. Modification indices were checked, and we accepted the suggestion that Variability be regressed on STMO, in line with its theoretically justified association (stochasticity consequent to short-term sociosexual interactions), as well as the empirically observed relationships between STMO and SDV in Study 2, and between STMO and H-SDV in both studies. No remaining modification indices were relatively large, thus further modifications were not accepted in order to limit potential overfitting.

## **Modifications made to instruments in Study 2**

### ***Sleep-wake stability***

The Sleep Timing Questionnaire is prefaced with the following, “Please answer in terms of a recent ‘normal average week,’ not one in which you traveled, vacationed or had family crises.” We added this prefatory note to the headings of multiple pages of the survey in order to better anchor the sleep consistency questions. “How stable (i.e., similar each night) are your GOOD NIGHT TIMES before a work day or school day?” could otherwise be potentially misinterpreted as asking about consistency between nights which are weeks or months apart. By reminding participants that we are interested in a “normal average week,” this increases the chances of receiving responses pertaining to night-to-night and morning-to-morning consistency over the course of a habitual week.

### ***Sleep need***

Sleep need is captured using the following question, "*Based on past experience, assuming you are not sleep deprived in any way*, how many hours of sleep

do you need per night *in order to feel fully rested the next day*?" The purpose of these modifications is to limit the amount of extraneous variance related to folk attitudes about how much sleep people “generally need.” Additionally, the modified verbiage accounts for the possibility of higher salience of sleep need on a sleep deprived day.

# **Results**

## ***Non-linear relationships between LHS and sleep duration in Study 1***

When LH K-Factor is plotted against habitual sleep duration using LOESS (locally estimated scatterplot smoothing), a roughly U-shaped fitted curve results (for conceptual clarity, slower LHS’s are lower on the y-axis), with the steepest portion of the curve reflecting the slowest LH strategies. Two confirmatory analyses were undertaken to test the apparent non-linear structure observed in the LOESS scatterplot of LH-K and habitual sleep duration. First, using the “Segmented” package in R [5], we performed segmented linear regression between LH K-Factor and habitual sleep duration using generalized linear models to estimate the point—if any—at which the slopes of the apparent curve intersect. This yielded an estimated breakpoint of 7.15 hrs (SE = .46). Compared to the null regression model, the breakpoint regression model had a significantly better fit (*p* = .029), with a reduction of 3.15 RSS from the null RSS of 118.18, and a loss of two degrees of freedom from 262 total observations. Second, we compared the null model to a 2^nd^ order polynomial model; the latter had a significantly better fit (*p* = .042), with a reduction of 1.86 RSS from the null RSS.

## ***Non-linear relationships between LHS and sleep duration in Study 2***

For the full cohort, the breakpoint model converged on 6.85 (SE = .64). When compared against the null model, the breakpoint model did not achieve a significantly better fit (*p* = .196). A 2^nd^ order polynomial model also did not achieve a significantly better fit (*p* = .162). R-squared values for the null, segmented, and 2^nd^ order polynomial regression models were 1.183 x 10^-4^, .011, and .007 respectively.

For the younger cohort, the breakpoint model converged on 7.17 (SE = .53). When compared against the null model, the breakpoint regression model had a significantly (*p* = .040) better fit, with a reduction of 3.07 RSS from the null RSS of 38.90, and a loss of two degrees of freedom from 79 total observations. A 2^nd^ order polynomial model also achieved a significantly better fit (p = .045), with a reduction of 1.96 RSS from the null RSS, and a loss of one degree of freedom from 79 total observations.

## ***Non-linear relationships between LHS and hedonic valuations in Study 1***

To account for the inherent noisiness of single-item measures, we parceled [6, 7] hedonic motivation and pleasure into a single variable titled “hedonic valuations for sleep” by z-scoring both variables and taking their arithmetic mean. Exploratory scatterplots between LHS and hedonic motivation/pleasure did not depict convincing models of higher order structures, with some ambiguity in the hedonic valuation parcel (Figure S6). As with previous confirmatory analyses, we used Segmented to check the hedonic valuation parcel for a breakpoint structure. While the model converged on an estimate of 0.39 (SE = .26), compared to the null linear model, the breakpoint model did not have a significantly better fit (*p* = .063). Then, we compared the null linear model to a 2^nd^ order polynomial model, which did not have a significantly better fit (*p* = .081). We also compared the null model against a 3^rd^ order polynomial model, which also did not have a significantly better fit (*p* = .052).

## ***Non-linear relationships between LHS and hedonic valuations in Study 2***

Using the aforementioned procedures we employed in Study 1, we compared a breakpoint model of hedonic valuation to the null linear model in the full cohort. The breakpoint model did not achieve a significantly better fit (*p* = .179). Both a 2^nd^ order polynomial model (*p* = .232) and a 3^rd^ order polynomial model (*p* = .457) also did not achieve a significantly better fit versus the null.

In the younger cohort, the breakpoint model did not achieve a significantly better fit than the linear model (*p* = .650). Both a 2^nd^ order polynomial model (*p* = .602) and a 3^rd^ order polynomial model (*p* = .650) also did not achieve a significantly better fit versus the null.

**References**

1. Kaplan BA, Amlung M, Reed DD, et al. Automating Scoring of Delay Discounting for the 21- and 27-Item Monetary Choice Questionnaires. *The Behavior analyst* 2016;**39**(2):293-304. doi: 10.1007/s40614-016-0070-9.

2. Stekhoven DJ. missForest: Nonparametric missing value imputation using random forest. *Astrophysics Source Code Library* 2015.

3. Dormann CF, Elith J, Bacher S, et al. Collinearity: a review of methods to deal with it and a simulation study evaluating their performance. *Ecography* 2013;**36**(1):27-46. doi: 10.1111/j.1600-0587.2012.07348.x.

4. DeYoung CG, Hasher L, Djikic M, et al. Morning people are stable people: Circadian rhythm and the higher-order factors of the Big Five. *Personality and Individual Differences* 2007;**43**(2):267-276. doi: https://doi.org/10.1016/j.paid.2006.11.030.

5. Muggeo VM. Segmented: an R package to fit regression models with broken-line relationships. *R news* 2008;**8**(1):20-25.

6. Little TD, Cunningham WA, Shahar G, et al. To parcel or not to parcel: Exploring the question, weighing the merits. *Structural equation modeling* 2002;**9**(2):151-173.

7. Little TD, Rhemtulla M, Gibson K, et al. Why the items versus parcels controversy needn’t be one. *Psychological methods* 2013;**18**(3):285.
