# Supplementary material for: Live fast, die young and sleep later: Life history strategy and human sleep behavior
Source: Evol Med Public Health. 2020 Dec 2;9(1):36–52. doi: 10.1093/emph/eoaa048 (PMC7953418; doi:10.1093/emph/eoaa048)
Supplement: eoaa048_Supplementary_Data [file eoaa048_supplementary_data.zip › EMPH-2020-089R2 Dishakjian et al Life History & Sleep Study 1-Survey Instruments-Corrected.pdf]

Please think of GOOD NIGHT TIME as the time at which you are finally in bed and trying to fall asleep.

1. On the night before a work day or school day, what is your **earliest** GOOD NIGHT TIME? (e.g. 7:25 pm)

2. On the night before a work day or school day, what is your **latest** GOOD NIGHT TIME? (e.g. 7:25 pm)

3. On the night before a work day or school day, what is your **usual** GOOD NIGHT TIME? (e.g. 7:25 pm)

4. How stable (i.e., similar each night) are your GOOD NIGHT TIMES before a work day or school day? (select one)

	0-15 minutes	16-30 minutes	31-45 minutes	46-60 minutes	61-75 minutes	76-90 minutes	91-105 minutes	106-120 minutes	2-3 hours	3-4 hours	over 4 hours	
**within**	○	○	○	○	○	○	○	○	○	○	○	**of one another**

5. What are the three biggest factors that NEGATIVELY affect **(delay)** your GOOD NIGHT TIME before a work or school day? (you may leave one or more fields blank)

Largest Detriment

Moderate Detriment

Smallest Detriment

Night before a day off

6. On the night before a day off (e.g. a weekend), what is your **earliest** GOOD NIGHT TIME? (e.g. 7:25 pm)

7. On the night before a day off (e.g. a weekend), what is your **latest** GOOD NIGHT TIME? (e.g. 7:25 pm)

8. On the night before a day off (e.g. a weekend), what is your **usual** GOOD NIGHT TIME? (e.g. 7:25 pm)

9. How stable (i.e., similar each night) are your GOOD NIGHT TIMES on a night before a day off (e.g. a weekend)? (select one)

	0-15 minutes	16-30 minutes	31-45 minutes	46-60 minutes	61-75 minutes	76-90 minutes	91-105 minutes	106-120 minutes	2-3 hours	3-4 hours	over 4 hours	
**within**	○	○	○	○	○	○	○	○	○	○	○	**of one another**

10. What are the three biggest factors that NEGATIVELY affect **(delay)** your GOOD NIGHT TIME before a day off? (you may leave one or more fields blank)

Largest Detriment

Moderate Detriment

Smallest Detriment

Morning before a typical work or school day

Please think of GOOD MORNING TIME as the time at which you finally get out of bed and start your day.

11. Before a work day or school day, what is your **earliest** GOOD MORNING TIME? (e.g. 7:25 am)

12. Before a work day or school day, what is your **latest** GOOD MORNING TIME? (e.g. 7:25 am)

13. Before a work day or school day, what is your **usual** GOOD MORNING TIME? (e.g. 7:25 am)

14. How stable (i.e., similar each morning) are your GOOD MORNING TIMES before a work day or school day? (select one)

	0-15 minutes	16-30 minutes	31-45 minutes	46-60 minutes	61-75 minutes	76-90 minutes	91-105 minutes	106-120 minutes	2-3 hours	3-4 hours	over 4 hours	
**within**	○	○	○	○	○	○	○	○	○	○	○	**of one another**

15. What are the three biggest factors that NEGATIVELY affect **(advance)** your GOOD MORNING TIME before a work or school day? (you may leave one or more fields blank)

Largest Detriment

Moderate Detriment

Smallest Detriment

Morning before a day off

16. Before a day off (e.g. a weekend), what is your **earliest** GOOD MORNING TIME? (e.g. 7:25 am)

17. Before a day off (e.g. a weekend), what is your **latest** GOOD MORNING TIME? (e.g. 7:25 am)

18. Before a day off (e.g. a weekend), what is your **usual** GOOD MORNING TIME? (e.g. 7:25 am)

19. How stable (i.e., similar each morning) are your GOOD MORNING TIMES before a day off (e.g. a weekend)? (select one)

	0-15 minutes	16-30 minutes	31-45 minutes	46-60 minutes	61-75 minutes	76-90 minutes	91-105 minutes	106-120 minutes	2-3 hours	3-4 hours	over 4 hours	
**within**	○	○	○	○	○	○	○	○	○	○	○	**of one another**

20. What are the three biggest factors that NEGATIVELY affect **(advance)** your GOOD MORNING TIME before a day off? (you may leave one or more fields blank)

Largest Detriment

Moderate Detriment

Smallest Detriment

Unwanted Wakefulness and Attitudes Toward Sleeping

These questions are about how much sleep you lose to unwanted wakefulness.

21. On most nights how long, on average, does it take you to fall asleep after you start trying?

minutes

22. On most nights, how much sleep do you lose, on average, from waking up during the night (e.g. to go to the bathroom, to attend to a crying baby, because of noise from neighbors or cars)?

[ ] minutes

23. Lastly, how many hours of sleep do you **need** per night? (How many hours would you sleep if you could hypothetically sleep as long as you needed to?)

I need [ ] hours and [ ] minutes of sleep per night.

24. Ignoring health considerations and biological requirements, how much do you enjoy the act of sleeping **compared to other activities**?

Sleeping is my least favorite activity	Sleeping is my favorite activity

25. If you could take a pill that would temporarily eliminate the need to sleep, how often would you take such a pill?

Never	Rarely	Regularly	Often	Always
○	○	○	○	○

Mini-k (Part 1/2)

Please indicate how strongly you agree or disagree with the following statements.

26. I can often tell how things will turn out.

Disagree Strongly	Disagree Somewhat	Disagree Slightly	Don't Know/Not Applicable	Agree Slightly	Agree Somewhat	Agree Strongly
○	○	○	○	○	○	○

27. I try to understand how I got into a situation to figure out how to handle it.

Disagree Strongly	Disagree Somewhat	Disagree Slightly	Don't Know/Not Applicable	Agree Slightly	Agree Somewhat	Agree Strongly
○	○	○	○	○	○	○

28. I often find the bright side to a bad situation.

Disagree Strongly	Disagree Somewhat	Disagree Slightly	Don't Know/Not Applicable	Agree Slightly	Agree Somewhat	Agree Strongly
○	○	○	○	○	○	○

29. I don’t give up until I solve my problems.

Disagree Strongly	Disagree Somewhat	Disagree Slightly	Don't Know/Not Applicable	Agree Slightly	Agree Somewhat	Agree Strongly
○	○	○	○	○	○	○

30. I often make plans in advance.

Disagree Strongly	Disagree Somewhat	Disagree Slightly	Don't Know/Not Applicable	Agree Slightly	Agree Somewhat	Agree Strongly
○	○	○	○	○	○	○

31. I avoid taking risks.

Disagree Strongly	Disagree Somewhat	Disagree Slightly	Don't Know/Not Applicable	Agree Slightly	Agree Somewhat	Agree Strongly
○	○	○	○	○	○	○

32. While growing up, I had a close and warm relationship with my biological mother.

Disagree Strongly	Disagree Somewhat	Disagree Slightly	Don't Know/Not Applicable	Agree Slightly	Agree Somewhat	Agree Strongly
○	○	○	○	○	○	○

33. While growing up, I had a close and warm relationship with my biological father.

Disagree Strongly	Disagree Somewhat	Disagree Slightly	Don't Know/Not Applicable	Agree Slightly	Agree Somewhat	Agree Strongly
○	○	○	○	○	○	○

34. I have a close and warm relationship with my own children.

Disagree Strongly	Disagree Somewhat	Disagree Slightly	Don't Know/Not Applicable	Agree Slightly	Agree Somewhat	Agree Strongly
○	○	○	○	○	○	○

35. I have a close and warm romantic relationship with my sexual partner.

Disagree Strongly	Disagree Somewhat	Disagree Slightly	Don't Know/Not Applicable	Agree Slightly	Agree Somewhat	Agree Strongly
○	○	○	○	○	○	○

Mini-k (Part 2/2)

36. I would rather have one than several sexual relationships at a time.

Disagree Strongly	Disagree Somewhat	Disagree Slightly	Don't Know/Not Applicable	Agree Slightly	Agree Somewhat	Agree Strongly
○	○	○	○	○	○	○

37. I have to be closely attached to someone before I am comfortable having sex with them.

Disagree Strongly	Disagree Somewhat	Disagree Slightly	Don't Know/Not Applicable	Agree Slightly	Agree Somewhat	Agree Strongly
○	○	○	○	○	○	○

38. I am often in social contact with my blood relatives.

Disagree Strongly	Disagree Somewhat	Disagree Slightly	Don't Know/Not Applicable	Agree Slightly	Agree Somewhat	Agree Strongly
○	○	○	○	○	○	○

39. I often **get** emotional support and practical help from my blood relatives.

Disagree Strongly	Disagree Somewhat	Disagree Slightly	Don't Know/Not Applicable	Agree Slightly	Agree Somewhat	Agree Strongly
○	○	○	○	○	○	○

40. I often **give** emotional support and practical help to my blood relatives.

Disagree Strongly	Disagree Somewhat	Disagree Slightly	Don't Know/Not Applicable	Agree Slightly	Agree Somewhat	Agree Strongly
○	○	○	○	○	○	○

41. I am often in social contact with my friends.

Disagree Strongly	Disagree Somewhat	Disagree Slightly	Don't Know/Not Applicable	Agree Slightly	Agree Somewhat	Agree Strongly
○	○	○	○	○	○	○

42. I often **get** emotional support and practical help from my friends.

Disagree Strongly	Disagree Somewhat	Disagree Slightly	Don't Know/Not Applicable	Agree Slightly	Agree Somewhat	Agree Strongly
○	○	○	○	○	○	○

43. I often **give** emotional support and practical help to my friends.

Disagree Strongly	Disagree Somewhat	Disagree Slightly	Don't Know/Not Applicable	Agree Slightly	Agree Somewhat	Agree Strongly
○	○	○	○	○	○	○

44. I am closely connected to and involved in my community.

Disagree Strongly	Disagree Somewhat	Disagree Slightly	Don't Know/Not Applicable	Agree Slightly	Agree Somewhat	Agree Strongly
○	○	○	○	○	○	○

45. I am closely connected to and involved in my religion.

Disagree Strongly	Disagree Somewhat	Disagree Slightly	Don't Know/Not Applicable	Agree Slightly	Agree Somewhat	Agree Strongly
○	○	○	○	○	○	○

46. If you go outside on a clear day and look up, what color should the sky usually be?

Snack	Antelope	Pink	Blue	FM Radio	Tuberculosis	Printer
○	○	○	○	○	○	○

Multidimensional Sociosexual Orientation Inventory

47. I can easily imagine myself being comfortable and enjoying “casual” sex with different partners.

Disagree Strongly	Disagree Somewhat	Disagree Slightly	Don't Know/Not Applicable	Agree Slightly	Agree Somewhat	Agree Strongly
○	○	○	○	○	○	○

48. I can imagine myself enjoying a brief sexual encounter with someone I find very attractive.

Disagree Strongly	Disagree Somewhat	Disagree Slightly	Don't Know/Not Applicable	Agree Slightly	Agree Somewhat	Agree Strongly
○	○	○	○	○	○	○

49. I could easily imagine myself enjoying one night of sex with someone I would never see again.

Disagree Strongly	Disagree Somewhat	Disagree Slightly	Don't Know/Not Applicable	Agree Slightly	Agree Somewhat	Agree Strongly
○	○	○	○	○	○	○

50. I am interested in maintaining a long-term romantic relationship with someone special.

Disagree Strongly	Disagree Somewhat	Disagree Slightly	Don't Know/Not Applicable	Agree Slightly	Agree Somewhat	Agree Strongly
○	○	○	○	○	○	○

51. I hope to have a romantic relationship that lasts the rest of my life.

Disagree Strongly	Disagree Somewhat	Disagree Slightly	Don't Know/Not Applicable	Agree Slightly	Agree Somewhat	Agree Strongly
○	○	○	○	○	○	○

52. Long-term romantic relationships are not for me.

Disagree Strongly	Disagree Somewhat	Disagree Slightly	Don't Know/Not Applicable	Agree Slightly	Agree Somewhat	Agree Strongly
○	○	○	○	○	○	○

53. During your entire life, with how many partners have you had sexual intercourse?

54. With how many partners have you had sexual intercourse within the past year?

55. With how many partners have you had sex on one and only one occasion?

Email Address for Raffle

To sign up for the optional raffle, please provide an email address below. You will have a chance to win up to **<u>$85</u>** (paid out using Zelle).

Should you choose to opt into this raffle, you will be asked to choose between prizes that vary in the amount and timing of payment. **The actual prize you receive, if you are chosen as the winner, will depend on these selections.**

Your email address, should you choose to provide it, will be **stored separately from the rest of your data**, and will not be used for any purpose other than to award the raffle's prize. As soon as the 24 hour period of data collection is over and the prize is distributed, **your email address will immediately be deleted**.

To sign up for the raffle, please enter your email address below. This email address will be used to award your prize, if you are chosen as the winner.

Discounting

By providing an email address, you have received a chance to win a monetary prize. **If you are selected as a winner, this prize will be randomly chosen from one of the following selections.**

To make sure that you get a reward you prefer, **you should assume that you are the winner**, and then make each choice as though it were the one you will win.

Pick ONE from EACH possible choice.

56.

○ $78 in the next 24 hours OR ○ $80 in 162 days

57.

- $80 in the next 24 hours OR $85 in 157 days

58.

- $67 in the next 24 hours OR $75 in 119 days

59.

- $69 in the next 24 hours OR $85 in 91 days

60.

- $55 in the next 24 hours OR $75 in 61 days

61.

- $54 in the next 24 hours OR $80 in 30 days

62.

- $41 in the next 24 hours OR $75 in 20 days

63.

- $33 in the next 24 hours OR $80 in 14 days

64.

- $31 in the next 24 hours OR $85 in 7 days

Thank You!
